# Collagen-mediated pro-tumorigenic MAPK activation drives stromal-immune reprogramming in solid cancers

**DOI:** 10.3389/fimmu.2026.1744126

**Published:** 2026-02-24

**Authors:** Junli Ding, Hongxin Lin, Hanfang Fan, Liangfeng Xu, Haihua Zhou, Huning Jiang, Zeyu Wang, Tiansong Xia, Yun Cai, Yichao Zhu, Junying Xu

**Affiliations:** 1 Departments of Oncology, The Affiliated Wuxi People’s Hospital of Nanjing Medical University, Wuxi, Jiangsu, China; 2 Wuxi Medical Center, Nanjing Medical University, Wuxi, Jiangsu, China; 3 Department of General Surgery, The First Affiliated Hospital with Nanjing Medical University, Nanjing, Jiangsu, China; 4 The First Clinical Medicine College, Nanjing Medical University, Nanjing, Jiangsu, China; 5 Department of Physiology, School of Basic Medical Sciences, Nanjing Medical University, Nanjing, Jiangsu, China; 6 Department of Gastroenterology, Sheyang County People’s Hospital, Yancheng, Jiangsu, China; 7 Department of General Surgery, The Affiliated Taizhou People’s Hospital of Nanjing Medical University, Taizhou, Jiangsu, China; 8 Taizhou School of Clinical Medicine, Nanjing Medical University, Taizhou, Jiangsu, China; 9 Central Laboratory, Jintan Affiliated Hospital of Jiangsu University, Changzhou, Jiangsu, China

**Keywords:** collagen deposition, M2 macrophage, MAPK, microvessel density, pan-cancer

## Abstract

**Introduction:**

Intratumoral collagen deposition is a hallmark of solid cancers and plays a critical role in shaping the tumor microenvironment (TME). This study aimed to characterize the clinical and molecular implications of collagen deposition and elucidate its role in modulating TME components and signaling pathways.

**Methods:**

In this research, we analyzed transcriptomic data from public databases and our in-house clinical samples to expore the correlation between collagen deposition and TME features. In addition, the findings were validated through in vitro and in vivo assays.

**Results:**

We found that high levels of intratumoral collagen deposition were related to poor clinical outcomes and advanced tumor stages in gastric cancer. Collagen deposition showed strong positive correlations with the abundance of vascular endothelial cells and M2-polarized macrophages, suggesting its role in promoting angiogenesis and immunosuppression. In addition, the correlations between collagen deposition and endothelial cells as well as M2-polarized macrophages were also confirmed in lung cancer. Moreover, pathway analysis revealed that collagen activated the MAPK signaling pathway, and *in vitro* and *in vivo* functional assays confirmed that collagen-mediated MAPK activation enhanced tumor cell invasion, angiogenesis, and M2 macrophage polarization.

**Conclusion:**

Our findings demonstrate that intratumoral collagen deposition is a key regulator of the TME in gastric cancer, promoting tumor progression through MAPK signaling pathway activation. These results demonstrate the promise of collagen as both a prognostic indicator and a therapeutic target, offering fresh perspectives on the underlying mechanisms of TME remodeling and tumor progression across various solid tumors.

## Introduction

Collagen is a predominant protein in the human body and the major constituent of connective tissue (1). It is a long fibrous protein composed of three intertwined protein chains in a helical structure, providing collagen with its unique strength and stability. Collagen serves various crucial functions in the body, including structural support, maintaining skin elasticity and firmness, aiding in wound healing, and supporting joint and cartilage health (2, 3). There are multiple types of collagen, such as type I, II, and III collagens (4). These different types of collagen have slightly varying distributions and functions in the human body. The role of collagen in tumors is a complex and diverse topic, dependent on the cancer type, stage, and microenvironment of the tumor. Increasing evidence suggests that collagen may act as significant roles in the growth, metastasis, and invasion of tumors, and the collagen surrounding tumors can impact the diffusion of drugs within tumor tissue, thus affecting treatment outcomes (5–8). While collagen is widely recognized as a pro-carcinogenic factor, it may also have inhibitory effects on tumor growth in certain circumstances.

The impact of collagen on the tumor microenvironment (TME) is a crucial factor in influencing tumor progression (9, 10). Collagen can influence the immune response of tumors by influencing the activity of immune cells in the TME. The size and density of collagen fibers directly affect the external resistance encountered by immune cells during migration, thereby influencing their speed and quantity entering the tissue. Hartmann et al. found that despite the upregulation of T cell chemokines CXCL10 or CXCL4 in pancreatic cancer, T lymphocyte infiltration in the tumor is still inhibited due to the presence of high-density collagen (11). Collagen receptors play an essential role in collagen-mediated immune effects. The LAIR protein family is an important collagen receptor. Several reports indicate that collagen in the extracellular matrix can directly inhibit the cytotoxic activity of T cells through the LAIR1 receptor (7, 12). Matrix stiffness has been shown to impact T cell migration, with T cell activation significantly inhibited when cultured on matrices with increased stiffness, including the inhibition of T cell proliferation and the expression of activity-related cytokines (13). Furthermore, increased stiffness in the stroma can inhibit the cGAS immune signaling pathway in tumor cells, promoting tumor immune evasion and indirectly leading to fewer infiltrating cytotoxic T cells (14). The evidence above suggests that collagen significantly impacts T cells, but the correlation and potential effects of collagen on different types of immune cells remain unclear.

In the current study, we aimed to characterize the clinical and molecular implications of intratumoral collagen deposition in gastric cancer and other cancer types. Using a combination of in silico analysis, in-house clinical cohorts, and functional assays, we sought to evaluate the correlation between collagen levels and different TME cell populations, identify the signaling pathways regulated by collagen, and elucidate the mechanisms by which collagen promotes tumor progression. Our findings provide new insights into the role of collagen in shaping the TME and highlight potential therapeutic targets for human cancers.

## Methods

### Collection of transcriptome data

Transcriptome data and clinical annotations for all tumor types were retrieved from The Cancer Genome Atlas (TCGA) via the University of California Santa Cruz (UCSC) Xena platform (https://xenabrowser.net/datapages/). Samples with documented overall survival (OS) information were specifically curated for subsequent analysis. Furthermore, a collection of publicly available immunotherapy datasets comprising transcriptome data from cancer patients receiving immunotherapy, including GSE173839 (15), GSE194040 (16), PRJEB25780 (17), GSE135222 (18), GSE78220 (19), and GSE176307 (20), was incorporated into the study. The MEDI4736 dataset was sourced from Dr. Lajos Pusztai’s study (21). By combining the above triple-negative breast cancer (TNBC) datasets (GSE173839, GSE194040, and MEDI4736), the “removeBatchEffect” function in the “limma” package (22) was used to remove batch effects.

### Bulk transcriptome data analysis

The collagen scores of each patient were calculated based on the collagen genes obtained from our previous study (8, 10) via the single-sample gene set enrichment analysis (ssGSEA) algorithm in the GSVA package. The full list of genes used for collagen score calculation was documented in **Supplementary Table 1**. The TME characteristics encompassed immunomodulators, levels of tumor-infiltrating immune cells and other stromal cells, and the presence of inhibitory immune checkpoints. The complete methodological framework for TME characterization has been previously described in our published work (23–26). Then, The TCGA and the PRJEB25780 cohorts were utilized to explore the correlations between Collagen scores and these TME characteristics.

### Single-cell RNA-sequencing data analysis

The single-cell RNA sequencing (scRNA-seq) datasets of 26 patients with primary gastric carcinoma were obtained from the GSE183904 dataset (27). We implemented rigorous quality control by excluding cells demonstrating either: (1) mitochondrial gene content >10%, (2) <200 detected genes, or (3) >5,000 detected genes (potential doublets). The “RunHarmony” function in the R package harmony (28) was used to mitigate the technical batch effects among individuals and experiments. Dimensionality reduction employed a dual-phase approach: first identifying 4,000 highly variable genes for principal component analysis (29), then selecting the top 30 principal components for t-SNE visualization (30). Cell clustering utilized the shared nearest neighbor clustering (SNN) graph-based algorithm (31) with modularity optimization at resolution=1. This analytical pipeline systematically resolved 96,162 high-quality cells into 35 transcriptionally distinct clusters, enabling comprehensive characterization of the gastric carcinoma ecosystem.

First, for each signature gene, we defined a background set comprising the 100 genes with the most comparable average expression levels. The expression value of each signature gene was then normalized by subtracting the mean expression of its background set. The migration and invasion scores were subsequently calculated by averaging these normalized values across all signature genes. Additionally, using the “AddModuleScore” function in Seurat, we quantified the activity of the MAPK signaling pathway, as well as the migration and extravasation potential of tumor cells, based on previously established gene signatures (32). In addition, the angiogenesis score in endothelial cells was also determined.

### Clinical samples

A total of 60 patients with gastric cancer were recruited from The Affiliated Wuxi People’s Hospital of Nanjing Medical University, following ethical approval (No. KY23176). Tumor tissue samples were obtained at surgery. All patients received standard post-surgical adjuvant therapy. Additionally, paraffin-embedded tissue microarrays containing 60 lung cancer samples were procured from the National Engineering Center for Biochip (Outdo Biotech, Shanghai, China) under approval No. SHYJS-CP-1601005.

### Histochemistry and immunohistochemistry analyses

Human paraffin-embedded tissues were sectioned at a thickness of 4 µm. The tissue sections were then subjected to Masson trichrome and immunohistochemical (IHC) staining. Standard operating procedures for Masson and IHC staining were as previously described (10, 33). Specifically, Masson staining was performed using a commercial Trichrome Stain Kit (Cat FH115100, FreeThinking, Nanjing, China) in accordance with the manufacturer’s protocol. Primary antibodies utilized were as follows: CD8 (prediluted, Cat. PA067, Abcarta, Suzhou, China), PD1 (prediluted, Cat. PA153, Abcarta), GZMB (1:3000 dilution, Cat. ab255598, Abcam, Cambridge, England), CD56 (prediluted, Cat. PA211, Abcarta), CD19 (prediluted, Cat. GT2128, GeneTech, Shanghai, China), CD86 (1:500 dilution, Cat. ab269587, Abcam), CD163 (prediluted, Cat. ab74604, Abcam), CD31 (1:2000 dilution, Cat. ab182981, Abcam), α-SMA (1:2000 dilution, Cat. ab124964, Abcam), PD-L1 (prediluted, Cat. GT2280, GeneTech), p-ERK (1:1000 dilution, Cat. 4370, Cell Signaling Technology, Danvers, USA), MLH1 (prediluted, Cat. GT2304, GeneTech), MSH2 (prediluted, Cat. GT2310, GeneTech), MSH6 (prediluted, Cat. GT2195, GeneTech), PMS2 (prediluted, Cat. GT2159, GeneTech), and IgG (1:100 dilution, Cat. ab172730, Abcam).

Masson staining evaluation involved determining positively stained area percentages and IHC analysis for most biomarkers were analyzed by determining the rate of positive cells using the HALO software (Albuquerque, NM, USA). Collagen deposition was classified as low (<10% area) or high (≥10% area), with a cutoff of 10% (10). PD-L1 staining was quantitatively assessed based on the combined positive score (CPS) criterion by two senior pathologists. For mismatch repair genes (MLH1, MSH2, MSH6, and PMS2) IHC analysis, two senior pathologists directly determined the positive and negative cases. **Supplementary Figure 1** exhibited the positive and negative staining for various markers in gastric cancer and lung cancer.

### Cell lines and cell culture

The HGC27 gastric carcinoma cell line was used as the primary disease-relevant model. The H1299 NSCLC cell line was selected to investigate the potential conservation of the collagen-MAPK signaling axis across different solid tumor types. Human cancer cell lines HGC27 (Cat. KGG3287-1) and NCI-H1299 (Cat. KGG3216-1) were purchased from KeyGEN (Nanjing, China). The vascular endothelial cell line HUVEC (Cat. SC0396) THP1 mononuclear cell line (Cat. SC0071) were purchased from YUCHI Biology (Shanghai, China). HGC27, NCI-H1299, and THP1 cells were cultured in RPMI-1640 medium supplemented with 10% FBS at 37 °C with 5% CO_2_. HUVEC cells were cultured in endothelial cell medium (Cat. 1001, ScienceCell, California, USA) at 37 °C with 5% CO_2_. Primary cancer-associated fibroblasts (CAFs) from gastric cancer tissues were extracted as previously described (34). All human cell lines were authenticated by short tandem repeat profiling, and all experiments were conducted in the absence of mycoplasma contamination. To differentiate THP-1 monocytic cells into macrophages, the cells were treated with 200 ng/mL of Phorbol 12-myristate 13-acetate (PMA, Cat. HY-18739). For ERK inhibition, 1 µM Ravoxertinib (Cat. T6511, TargetMol, Shanghai, China) was used.

### Western blotting analysis and immunofluorescence

Total protein was extracted from human cells using lysis buffer, followed by SDS-PAGE and Western blotting according to standard protocols. The following primary antibodies were used: p-MEK (Cat. 9154, Cell Signaling Technology; 1:1000), MEK (Cat. A4868, Abclonal; 1:1000), p-ERK (Cat. 9102, Cell Signaling Technology; 1:1000), ERK (Cat. A4782, Abclonal; 1:1000), and Vinculin (Cat. 66305-1-Ig, ProteinTech; 1:5000). Vinculin was used as a loading control for normalization. The subcellular localization of ERK was examined by immunofluorescence using a specific antibody (Cat. A4782, Abclonal; 1:500), and images were acquired with a fluorescence microscope.

### *In vitro* macrophage polarization assay

To induce M2 polarization, THP-1-derived macrophages were stimulated with 20 ng/ml IL-4 (Cat. KGD1203, KeyGEN) for 24 hours. The polarization status was then assessed by flow cytometry, measuring the expression of the M1 marker CD86 and the M2 marker CD163. The following antibodies were used: APC anti-CD86 (Cat. PE-65165, ProteinTech) and PE anti-CD163 (Cat. APC-65169, ProteinTech).

### Collagen coating and functional assays

Prior to cell culture, plates were pre-coated with Type I collagen (Cat. A1048301, Gibco, Thermo Fisher Scientific, MA, USA) using a method standardized in our laboratory and consistent with the manufacturer’s protocol (35). The final concentration used *in vitro* assays was 10 μg/cm^2^. Cells were seeded onto culture plates for collagen stimulation for 24 hours.

Cell proliferation was assessed by CCK-8 assay. Briefly, cells were seeded in 96-well plates at 5 × 10^3^ cells/mL (100 μL/well) and cultured for 24 hours. After adding 10 μL of CCK-8 reagent (Cat. KGA9310, KeyGEN), the plates were incubated for 1 hour, and the absorbance at 450 nm was measured with a microplate reader. Cell migration and invasion were evaluated using Transwell chambers, uncoated or pre-coated with Matrigel, respectively. Following digestion with 0.25% trypsin, 5 × 10^4^ cells in 200 μL serum-free medium were plated in the upper chamber, while the lower chamber was filled with 600 μL medium containing 10% FBS. After 24 hours, cells that had traversed the membrane were fixed with 4% paraformaldehyde, stained with 0.2% crystal violet, and quantified by counting three random 100× fields. Apoptosis was analyzed using an Annexin V-FITC/PI Kit (Cat. KGA1102, KeyGEN) per the manufacturer’s instructions. In accordance with the kit’s recommendation, only early apoptotic cells were enumerated to avoid overlap with late apoptotic and necrotic populations. For the tube formation assay, HUVECs (3 × 10^4^ cells/well) were seeded onto a solidified layer of Matrigel (50 µL/well, Cat. KGL5101, KeyGEN) in a 96-well plate and incubated for 6 hours. Tube formation was assessed by counting tubular structures from three random 100× microscopic fields.

### Tumor-bearing mouse model and drug treatment

All experimental procedures used 5-week-old male 615 mice supplied by Hangzhou Ziyuan Animal Co., Ltd. (Hangzhou, China). Animals were housed in a specific pathogen-free environment with a 12-h light/dark cycle, controlled temperature (20–24 °C), and ad libitum access to food and water. All mouse studies were approved by the Laboratory Animal Ethics Committee of Wuxi People’s Hospital (No. DL2024008). The murine gastric cancer cell line MFC (Cat. KGG2227-1, KeyGEN Biotech, Nanjing, China) and fibroblast cell line 3T3 (Cat. KGG1305-1, KeyGEN Biotech) were cultured in Dulbecco’s minimum essential medium supplemented with 10% FBS at 37 °C with 5% CO_2_. All cell lines were free from mycoplasm. A mouse model of gastric cancer was established by subcutaneous inoculation of approximately 5 × 10^6^ tumor cells, either alone or pre-mixed with fibroblasts at a 1:1 ratio, into each 615 mouse. Tumor dimensions were measured every 2–3 days using calipers, and the volume was calculated as (length × width^2^)/2. When the average volume of control tumors (without fibroblasts) reached approximately 100 mm^3^, mice bearing tumors derived from the cell-fibroblast mixture were randomly divided into two groups. One group received daily oral gavage of phosphate-buffered saline (PBS) as a vehicle control, while the other was treated with 20 mg/kg Ravoxertinib (Cat. T6511, TargetMol) administered orally each day.

On day 21 post-treatment initiation, tumors were excised from anesthetized animals, documented, and weighed. The harvested tumor tissues were then processed for subsequent Masson and IHC staining. Primary antibodies utilized were as follows: α-SMA (1:2000 dilution, Cat. ab124964, Abcam), p-ERK (1:1000 dilution, Cat. 4370, Cell Signaling Technology), Ki67 (1:1000 dilution, Cat. ab15580, Abcam), CD163 (1:500 dilution, Cat. ab182422, Abcam), and CD31 (1:2000 dilution, Cat. ab182981, Abcam).

### Statistical analysis

All statistical analyses and graphical representations were performed using R language (v4.0.2) and GraphPad Prism (v6.0). Group differences were analyzed with the Student’s t-test or Mann-Whitney test for two groups, and with one-way ANOVA or Kruskal-Wallis test followed by multiple comparisons for multiple groups. Associations between categorical variables were evaluated using the chi-square or Fisher’s exact test, while correlations were evaluated by Pearson’s or Spearman’s method. The diagnostic performance of candidate biomarkers was assessed by receiver-operating characteristic (ROC) curve analysis, with the area under the curve (AUC) quantifying their specificity and sensitivity. For survival data, the prognostic significance of categorical variables was assessed using the log-rank test. A P-value of less than 0.05 was considered statistically significant.

## Results

### Clinical relevance of collagen deposition in gastric cancer

**Figure 1:**
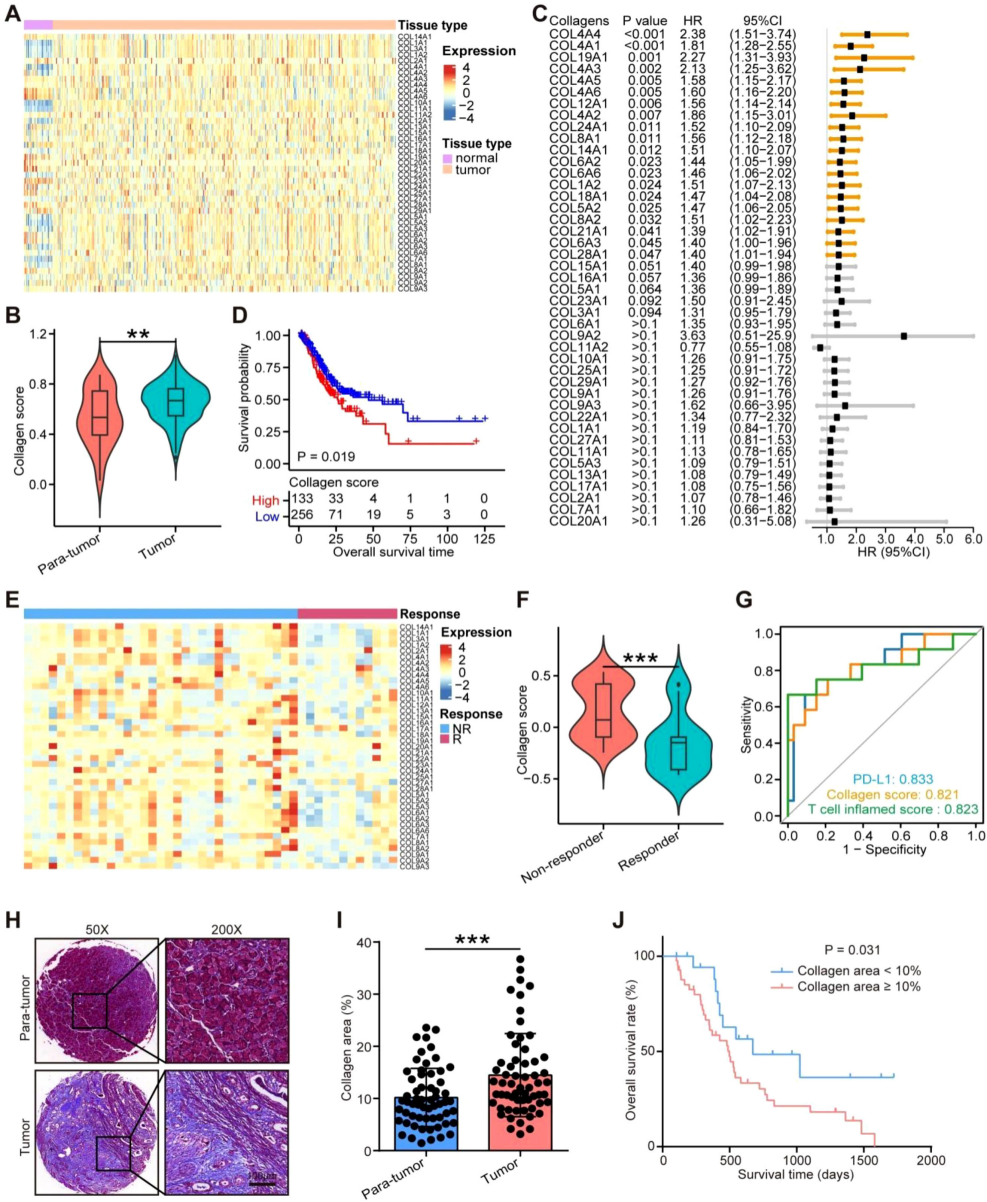
Clinical relevance of collagen deposition in gastric cancer. **(A)** Expression levels of various collagen molecules in tumor and para-tumor tissues from the TCGA-STAD cohort. **(B)** Collagen score in tumor and para-tumor tissues from the TCGA-SATD cohort. Significance was calculated using the student t test. ***: P < 0.001. **(C)** Prognostic values of various collagen molecules in gastric cancer in the TCGA-STAD cohort. Significance was calculated using the log-rank test. **(D)** Prognostic values of collagen score in the TCGA-STAD cohort. Significance was calculated using the log-rank test. **(E)** Expression levels of various collagen molecules in gastric cancer tissues from responders and non-responders to immunotherapy in the PRJEB25780 cohort. **(F)** Collagen score in gastric cancer tissues from responders and non-responders to immunotherapy in the PRJEB25780 cohort. Significance was calculated using the student t test. ***: P < 0.001. **(G)** ROC analysis of collagen score, PD-L1 expression, and T inflamed score in predicting immunotherapy response in gastric cancer in the PRJEB25780 cohort. **(H, I)** Representative images uncovering collagen distribution in tumor and para-tumor tissues in the in-house gastric cancer cohort and quantitative analysis. Total original magnification, 200×. Bar = 100 µm. Significance was calculated using the student t test. ***: P < 0.001. **(J)** Prognostic values of collagen level in the in-house gastric cancer cohort. Significance was calculated using the log-rank test. **P<0.01.

Given that the exact clinical value of collagen deposition has not been well defined, we checked the differential expressions and prognostic values of various collagens in gastric cancer, one of cancer types with intermediate collagen deposition levels in solid tumors (10). In the TCGA-STAD cohort, compared with para-tumor tissues, most collagen molecules were notably increased in cancer tissues (**Figure 1A**), and was correspondingly higher in tumor samples (**Figure 1B**). Nearly 50% of these collagen molecules were associated with unfavorable clinical outcomes (**Figure 1C**). Furthermore, a composite collagen score, derived from all collagen molecules, was significantly elevated in tumors and also predicted poorer prognosis (**Figure 1D**). Moreover, collagen deposition was associated with advanced T stage and TNM stage (**Supplementary Figure 2**). We also examined the predictive value of collagen deposition in gastric cancer in the PRJEB25780 cohort. As shown in the results, most collagen molecules were up-regulated in tumor tissues from these non-responders compared with responders to immunotherapy (**Figure 1E**). Interestingly, collagen score was significantly related to the response to immunotherapy and the predictive value of collagen score was even close to PD-L1 expression and T cell inflamed score (**Figures 1F, G**). We also validated these results in the in-house cohorts. We detected the total collagen level by the Masson staining, and the results exhibited that total collagen level was remarkably increased in tumor tissues (**Figures 1H, I**). In addition, high collagen level was linked with poor prognosis in the validated cohort (**Figure 1J**). Overall, collagen was significantly accumulated in gastric tumor tissues and associated with critical clinical outcomes.

### Correlations between collagen and microenvironmental cell fractions in gastric cancer

**Figure 2:**
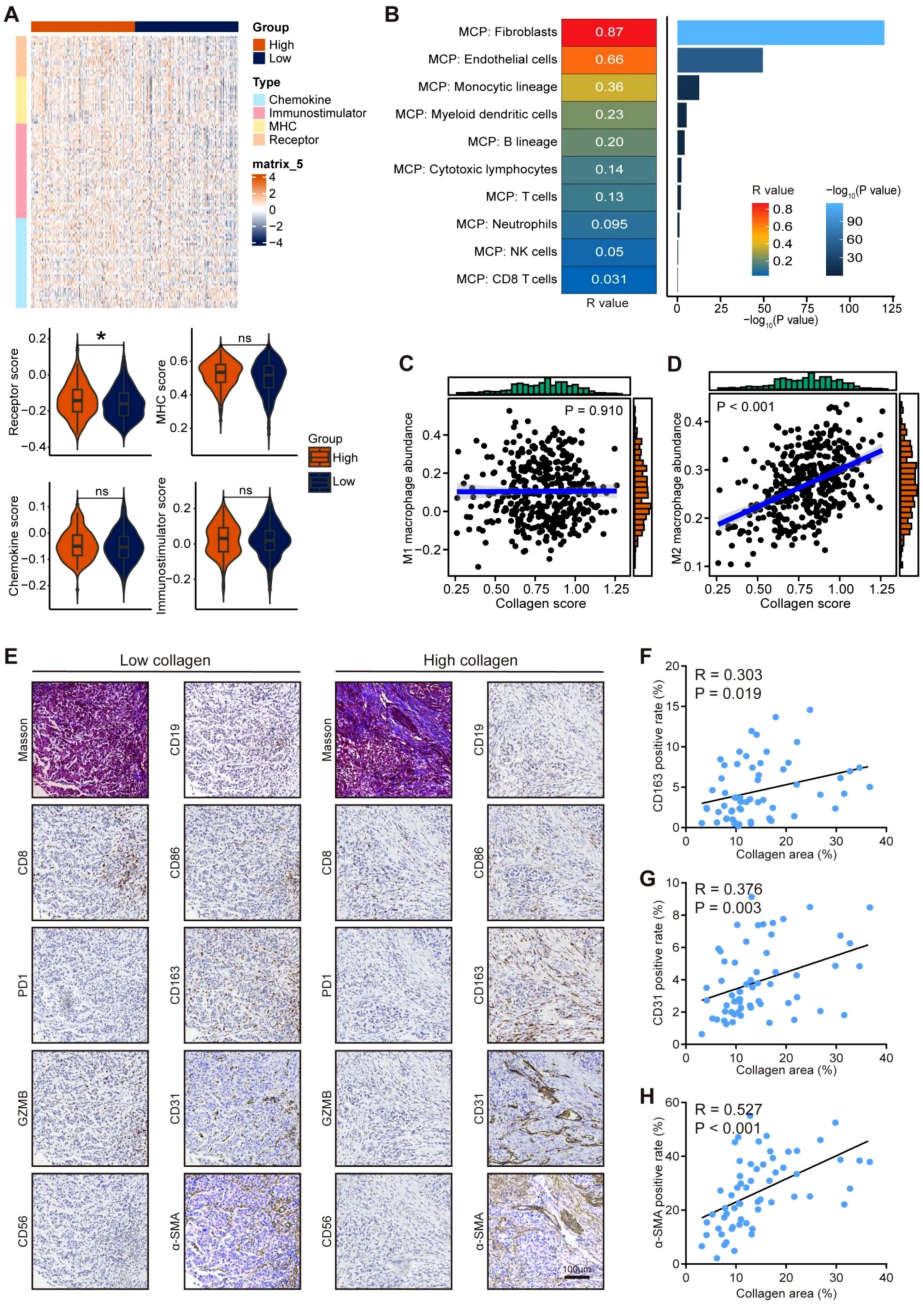
Correlations between collagen deposition and cell fractions in gastric cancer. **(A)** Heatmap showing the expression of MHC molecules, immunostimulators, chemokines and receptors in low and high collagen scores groups and GSVA analysis in the TCGA-STAD cohort. Significance was calculated using the student t test. ns: non-significance, *: P < 0.05. **(B)** Correlations between collagen score and various TME cells in the TCGA-STAD cohort. Significance was calculated using the Pearson test. **(C, D)** Correlations between collagen score and M1, M2 macrophages in the TCGA-STAD cohort. Significance was calculated using the Pearson test. **(E)** Representative images uncovering fractions various of cell types, including cytotoxic T cells (CD8), exhausted T cells (PD1), activated T cells (GZMB), NK cells (CD56), B cells (CD19), M1 macrophages (CD86), M2 macrophages (CD163), endothelial cells (CD31), fibroblasts (α-SMA), in tumor tissues with low and high collagen levels in the in-house gastric cancer cohort. Total original magnification, 200×. Bar = 100 µm. **(F–H)** Correlations between collagen levels with M2 macrophages (CD163), endothelial cells (CD31), and fibroblasts (α-SMA) rates in the in-house gastric cancer cohort. Significance was calculated using the Pearson test.

Next, we checked the potential correlations between collagen deposition and the features of the TME. Although expression patterns of MHC molecules, immunostimulators, chemokines, and their receptors did not differ markedly between gastric cancer tissues with low versus high collagen scores (**Figure 2A**), we further investigated the cellular composition of the TME given its inherent complexity. Using the MCP-counter algorithm, we estimated the relative abundance of immune and stromal cell populations in each sample. Pearson correlation analyses demonstrated significant associations between collagen scores and specific TME cell subsets. Collagens score was positive correlated with fibroblasts, endothelial cells, and monocytic lineage (**Figure 2B**). Subsequent analysis of macrophage polarization revealed a specific positive correlation with the M2 subset, but not with M1 macrophages (**Figures 2C, D**). Moreover, the results from the PRJEB25780 dataset also confirmed the positive correlations between collagen score between fibroblasts, endothelial cells, and M2 macrophage (**Supplementary Figures 3A–D**). For further validation, we conducted Masson staining and IHC analysis of various cell subsets (**Figure 2E**), illustrating positive correlations between collagen and M2 macrophages, endothelial cells, and fibroblasts (**Figures 2F–H**, **Supplementary Figure 4**). To conclusion, these results uncovered the positive correlations between collagen and M2 macrophages, endothelial cells, and fibroblasts, which might account for the oncogenic role of collagen in cancer.

### Correlations between collagen and crucial molecular events in gastric cancer

**Figure 3:**
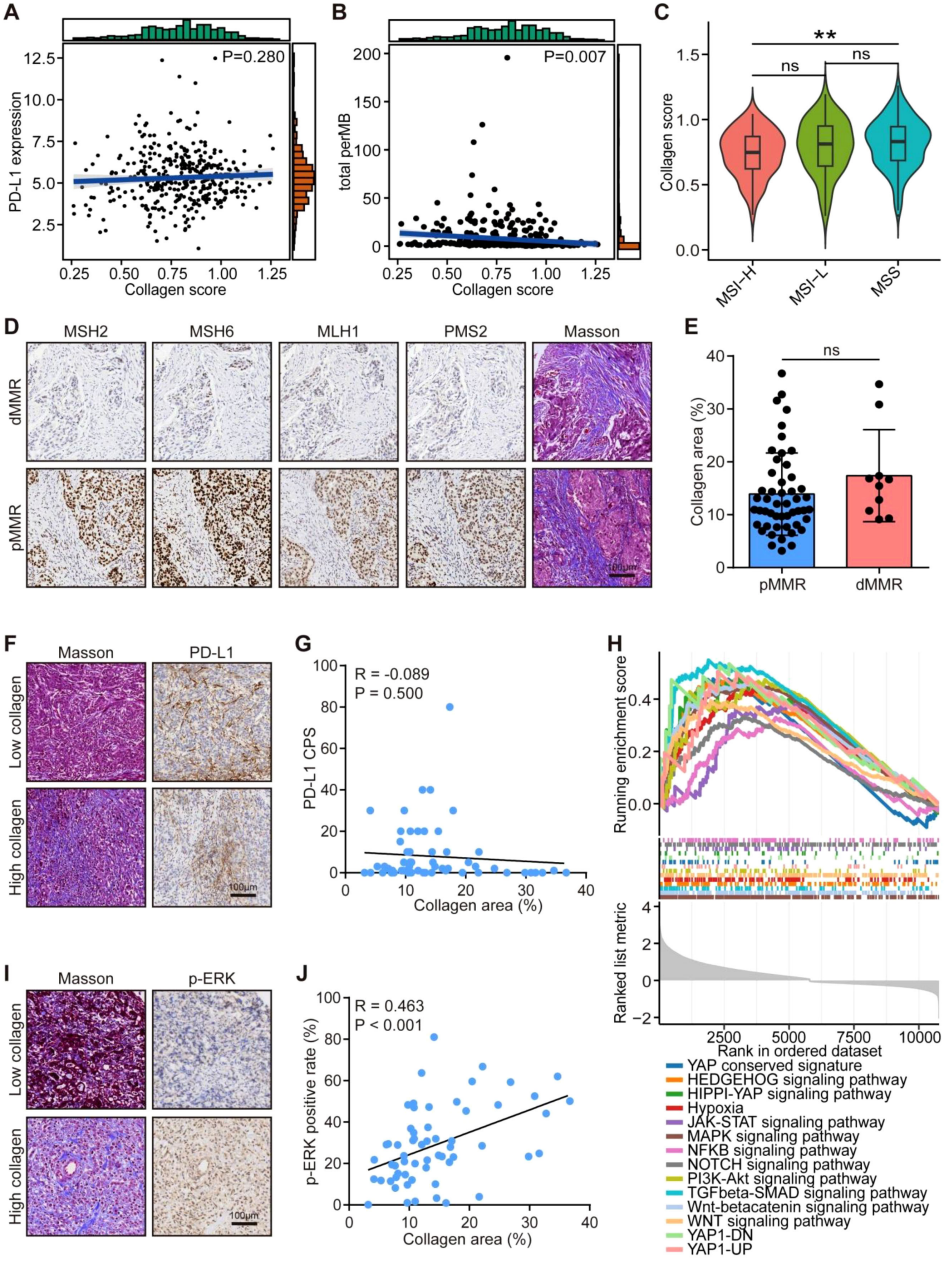
Correlations between collagen deposition and molecular events in gastric cancer. **(A, B)** Correlations between collagen score with PD-L1 expression and TMB levels in the TCGA-STAD cohort. Significance was calculated using the Pearson test. **(C)** Difference in collagen scores in gastric cancer with various MSI status. Significance was calculated using the one-way analysis of variance test with multiple comparisons. ns: non-significance, **P < 0.01. **(D, E)** Representative images uncovering collagen distribution in tumor tissues with different MMR status in gastric cancer and quantitative analysis. Total original magnification, 200×. Bar = 100 µm. Significance was calculated using the student t test. ns: non-significance. **(F, G)** Correlation between collagen levels with PD-L1 expression. Based on Masson staining, samples were classified as High collagen (≥10% stained area) or Low collagen (<10%). Total original magnification, 200×. Bar = 100 µm. Significance was calculated using the Pearson test. **(H)** GSEA analysis of correlations between collagen score with the activities of various molecular pathways in the TCGA-STAD cohort. **(I, J)** Correlation between collagen levels with p-ERK expression. Based on Masson staining, samples were classified as High collagen (≥10% stained area) or Low collagen (<10%). Total original magnification, 200×. Bar = 100 µm. Significance was calculated using the Pearson test.

We also evaluated the associations between collagen and crucial molecular events in gastric cancer. Although collagen score was associated with the immunotherapeutic responses, collagen scores exhibited no significant correlations with PD-L1 expression and tumor mutational burden (TMB) in the TCGA dataset (**Figures 3A, B**). Also, collagen levels were not significantly various across microsatellite instability (MSI) status subgroups in the TCGA dataset (**Figure 3C**). In the PRJEB25780 dataset, collagen score were not associated with most immune checkpoints and PD-L1 expression (**Supplementary Figures 5A–D**). In the in-house cohort, no significant differences in collagen distribution were observed based on mismatch repair (MMR) status (**Figures 3D, E**). No obvious correlation was found between collagen distribution and PD-L1 expression as well (**Figures 3F, G**). To understand the potential molecular mechanisms of collagen in cancer, GSEA linked high collagen scores to activation of ERK signaling pathways (**Figure 3H**, **Supplementary Table 2**), consistent with a strong positive correlation between collagen levels and p-ERK expression in the in-house cohort (**Figures 3I, J**). These findings underscore collagen deposition as a key regulator of both immunosuppressive TME remodeling and oncogenic signaling in cancer.

### Pan-cancer exploration of correlations between collagen and immune & molecular features

**Figure 4:**
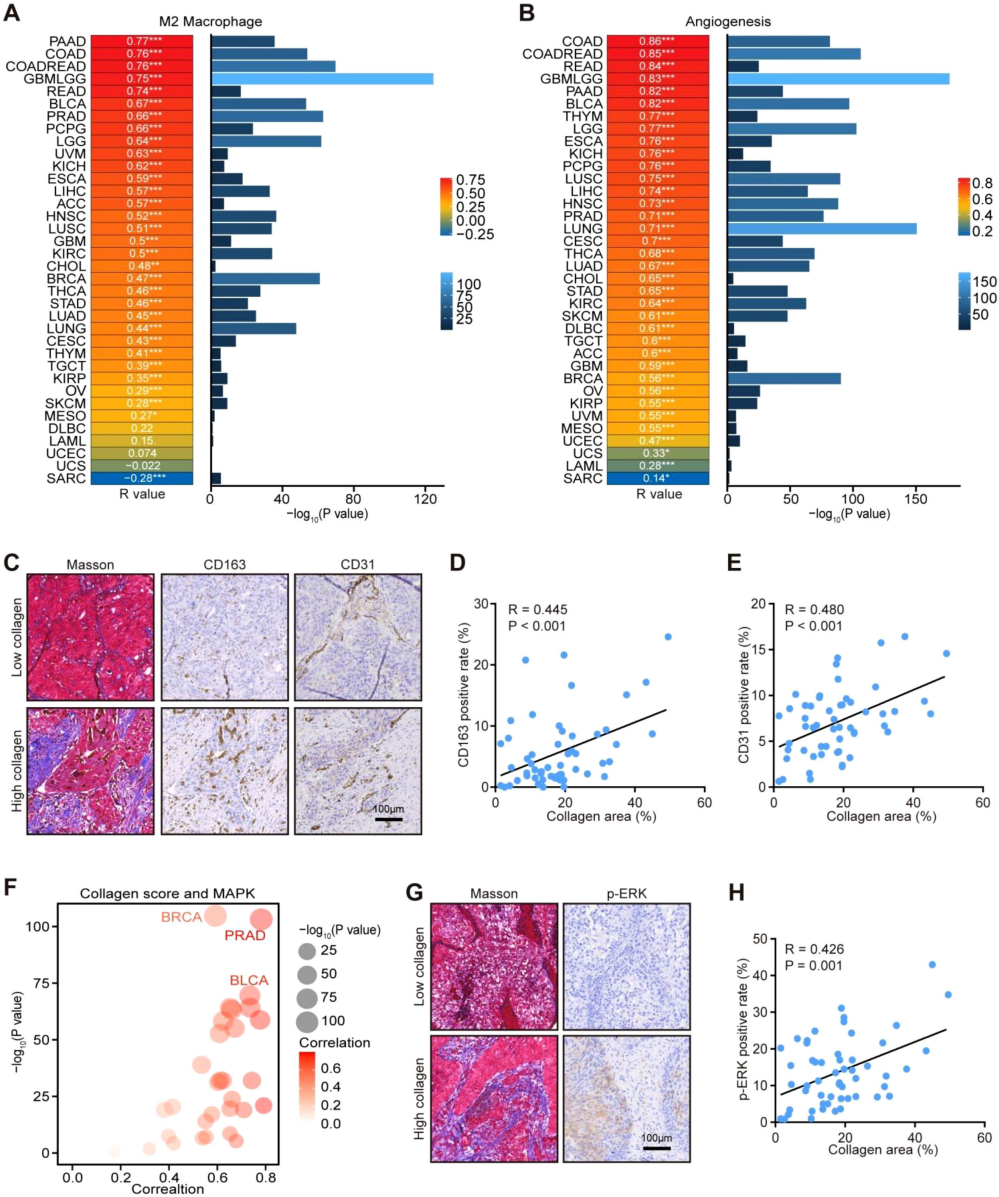
Pan-cancer analysis of correlations between collagen deposition and M2 macrophage & angiogenesis. **(A)** Correlations between collagen score and M2 macrophage abundance in pan-cancer from the TCGA database. Significance was calculated using the Pearson test. **(B)** Correlations between collagen score and angiogenesis level in pan-cancer from the TCGA database. Data obtained from the TCGA dataset. Significance was calculated using the Pearson test. **(C–E)** Representative images uncovering α-SMA and CD31 expression in tumor tissues in lung cancer with low and high collagen deposition and quantitative analysis. Total original magnification, 200×. Bar = 100 µm. Significance was calculated using the Pearson test. Based on Masson staining, samples were classified as High collagen (≥10% stained area) or Low collagen (<10%). **(F)** Pan-cancer analysis of the correlations between collagen and the MAPK signaling pathway scores from the TCGA database. Significance was calculated using the Pearson test. **(G, H)** Representative images uncovering p-ERK expression in tumor tissues in lung cancer with low and high collagen deposition and quantitative analysis. Total original magnification, 200×. Bar = 100 µm. Significance was calculated using the Pearson test. *P<0.05, **P<0.01, ***P<0.001.

Based on the above findings in gastric cancer, we further validated the correlations between collagen and M2 macrophage, angiogenesis, and the MAPK signaling pathways in other solid cancers. In the TCGA database, we found that collagen score was positively correlated with M2 macrophage level and angiogenesis activity in most solid cancer types, especially carcinomas (**Figures 4A, B**). In addition, one in-house lung cancer cohort was used for validation. Notably, the collagen levels were positively related to the positive rates of both CD163 and CD31, the markers for M2 macrophage and vascular endothelial cells (**Figures 4C–E**). Also, we explored the correlation between collagen and the activity of the MAPK signaling pathway, and the results exhibited that collagen was positively correlated with the activity of the MAPK signaling pathway, especially lung cancer (**Figure 4F**). Moreover, these results were also validated in the in-house lung cancer cohort by detecting the total collagen level by the Masson staining and the positive rate of p-ERK by the IHC staining (**Figures 4G, H**). The predictive value of collagen for immunotherapy was also checked in more solid cancer types. The results revealed that high collagen score predicted the resistance to immunotherapy in most cancer types suitable for immunotherapy, including melanoma, urothelial cancer, triple-negative breast cancer, and non-small cell lung cancer (**Supplementary Figures 6A–D**). Overall, the correlations between collagen and immuno-suppressive TME and immunotherapeutic resistance were conserved in pan-cancer.

### Oncogenic role of collagen was dependent on the MAPK signaling pathway in various cells

**Figure 5:**
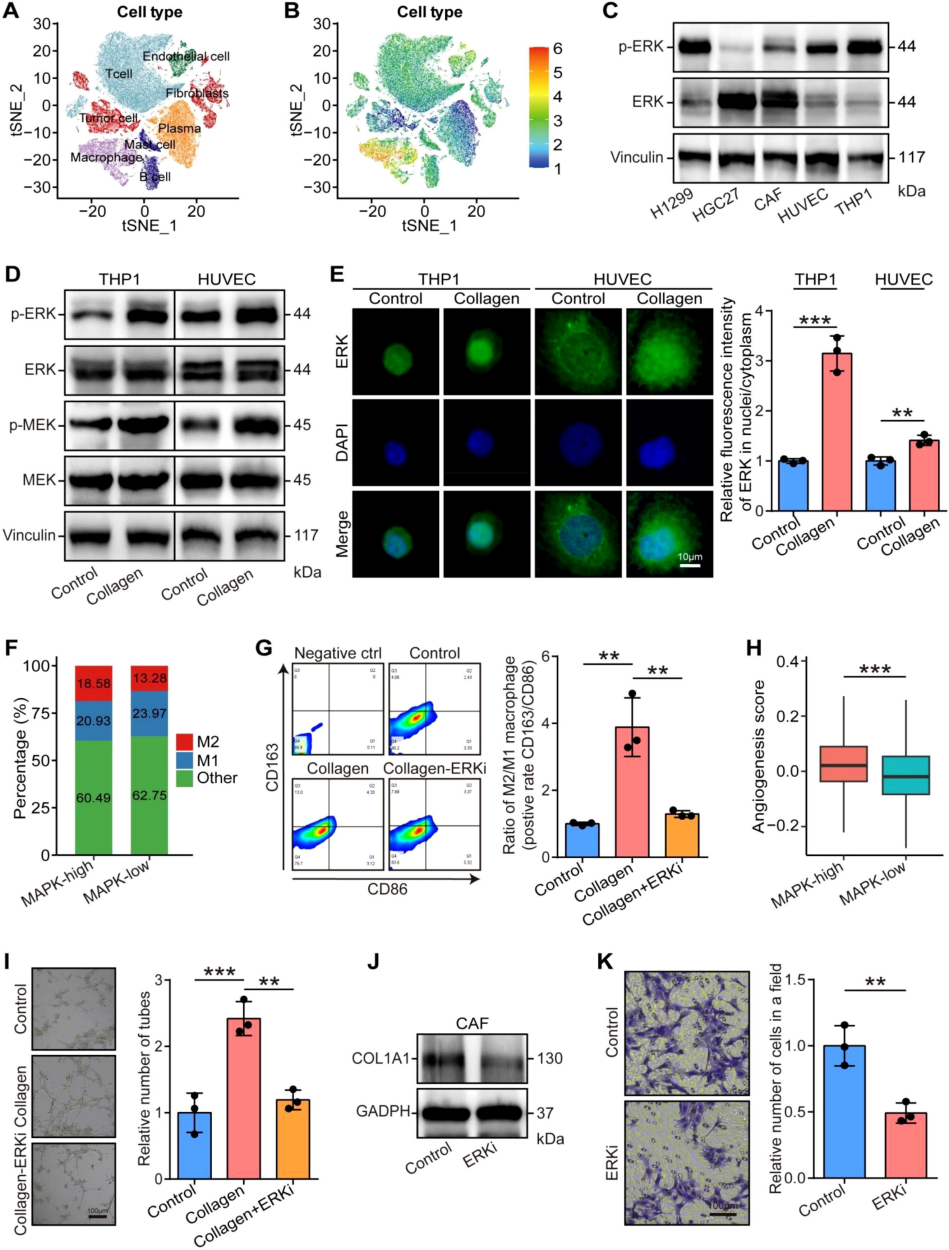
Cellular roles of collagen-mediated MAPK signaling pathway in stromal cells. **(A)** t-SNE visualization of single cells passed quality controls with different cell subtypes. **(B)** t-SNE visualization of the activities of MAPK signaling pathway in different cell subtypes. **(C)** Total and phosphorylated expression of ERK in various cell types, including tumor cells, CAFs, macrophages, and endothelial cells. **(D)** Activated effects of collagen on ERK and MEK in THP1 and HUVEC cells. **(E)** Effects of collagen on the nuclear translocation of ERK in THP1 and HUVEC cells and quantitative analysis. Bar = 10 µm. Significance was calculated using the student t test. **P<0.01, ***P<0.001. **(F)** Percentage of macrophage subtypes in low and high MAPK activities in the scRNA-seq dataset. **(G)** Effects of collagen and ERK inhibition on macrophage polarization. Significance was calculated using the one-way analysis of variance test with multiple comparisons. **P<0.01. **(H)** Angiogenesis score in endothelial cells in low and high MAPK activities in the scRNA-seq dataset. Significance was calculated using the student t test. ***P<0.001. **(I)** Effects of collagen and ERK inhibition on vascularization of endothelial cells. Total original magnification, 200×. Bar = 100 µm. Significance was calculated using the one-way analysis of variance test with multiple comparisons. **P<0.01, ***P<0.001. **(J)** Effects of ERK inhibition on collagen I expression in CAFs. **(K)** Effects of ERK inhibition on migration of CAFs. Total original magnification, 200×. Bar = 100 µm. Significance was calculated using the student t test. **P<0.01.

Given the tight correlations between collagen deposition and the MAPK signaling pathway, we speculated that collagen-mediated tumor cells aggressiveness, macrophage M2 polarization, and angiogenesis were based on the activity of the MAPK signaling pathway. We first checked the activities of the MAPK signaling pathway in various cell types in tumor tissues, and the results showed that the MAPK signaling pathway was conserved in various cells, especially macrophages and vascular endothelial cells (**Figures 5A, B**). We also validated the expression and phosphorylated levels of ERK, the critical molecule of the MAPK signaling pathway, which was expressed in all cell types, including tumor cells H1299 and HGC27, CAFs, endothelial cells HUVEC, and macrophage THP1 (**Figure 5C**). Next, we checked the effects of collagen on the MAPK signaling pathway in stromal cells, including HUVEC and THP1 cells. Collagen enhanced the phosphorylated levels of MEK and ERK and promoted the nuclear translocation of ERK in HUVEC and THP1 cells (**Figures 5D, E**). The scRNA-seq analysis indicated that macrophages with high MAPK activities exhibited increased M2 phenotype (**Figure 5F**). Additionally, collagen stimulation significantly elevated p-p38 levels in H1299 and HGC27 cells, consistent with earlier observations. By contrast, no notable p-p38 activation was detected in THP-1 or HUVEC cells following collagen treatment (**Supplementary Figure 7**), further support the cell-type-specific activation of p38 and reinforce the conclusion that the classical MEK/ERK pathway acts as the common downstream signaling cascade activated by collagen across the relevant cellular models. *In vitro* assays indicated that collagen promoted the M2 polarization of THP1 cells, but the ERK inhibitor Ravoxertinib could reversed the promoting effects (**Figure 5G**). Moreover, the MAPK activities were associated with angiogenesis activities in the scRNA-seq analysis and the ERK inhibitor Ravoxertinib inhibited the collagen-mediated angiogenesis of HUVEC cells (**Figures 5H, I**). Given that the collagen is essentially derived from CAFs, we also evaluated the cellular effects of ERK inhibition on CAFs. The results showed that Ravoxertinib inhibited collagen expression and the migration of CAFs (**Figures 5J, K**).

**Figure 6:**
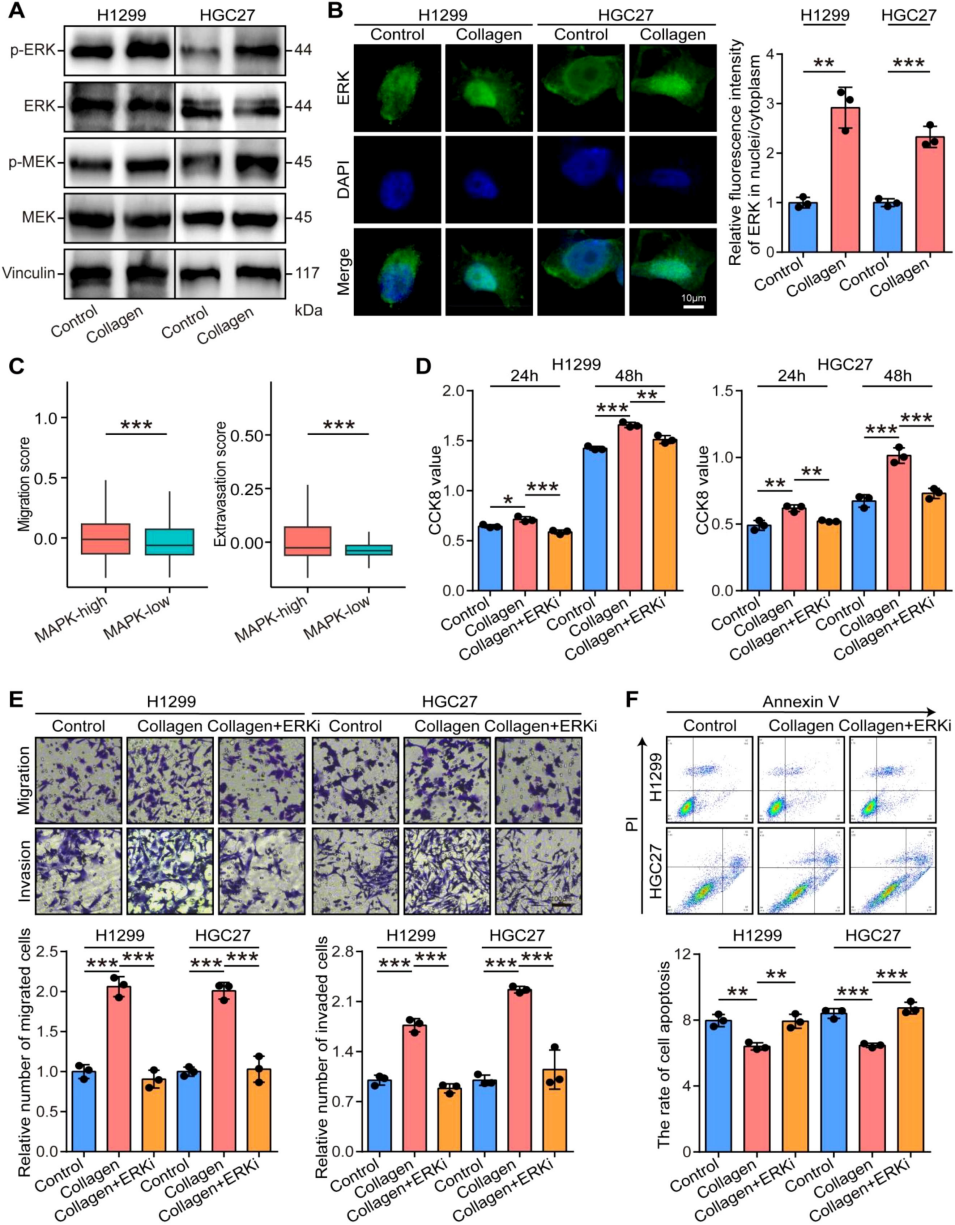
Cellular roles of collagen-mediated MAPK signaling pathway in tumor cells. **(A)** Activated effects of collagen on ERK and MEK in H1299 and HGC27 cells. **(B)** Effects of collagen on the nuclear translocation of ERK in H1299 and HGC27 cells and quantitative analysis. Bar = 10 µm. Significance was calculated using the student t test. **P<0.01, ***P<0.001. **(C)** Migration and extravasation scores in tumor cells in low and high MAPK activities in the scRNA-seq dataset. Significance was calculated using the student t test. ***P<0.001. **(D)** The proliferative capacity of control, collagen-induced, and ERKi-rescured tumor cells was examined at 24 and 48 h by CCK-8 assay. Significance was calculated using the one-way analysis of variance test with multiple comparisons. *P<0.05, **P<0.01, ***P<0.001. **(E)** The migratory and invasive capacities of control, collagen-induced, and ERKi-rescured tumor cells were examined at 24 h by Boyden chamber assay. Total original magnification, 200×. Bar = 100 µm. Significance was calculated using the one-way analysis of variance test with multiple comparisons. ***P < 0.001. **(F)** The apoptosis of control, collagen-induced, and ERKi-rescured tumor cells were examined by flow cytometry. Significance was calculated using the one-way analysis of variance test with multiple comparisons. **P < 0.01, ***P < 0.001.

In addition, we also evaluated the effects of collagen on the MAPK signaling pathway in tumor cells. We found that collagen enhanced the phosphorylated levels of MEK and ERK and promoted the nuclear translocation of ERK in H1299 and HGC27 tumor cells (**Figures 6A, B**). Based on the scRNA-seq analysis, we found that the activities of the MAPK signaling pathway were related to the proliferation and aggressiveness potentials in tumor cells (**Figure 6C**). Further *in vitro* assays indicated that collagen promoted the proliferation, migration, and invasion of H1299 and HGC27 tumor cells, but the ERK inhibitor Ravoxertinib could reversed the promoting effects (**Figures 6D, E**). In addition, the ERK inhibitor Ravoxertinib also enhanced the collagen-mediated inhibition of tumor cells apoptosis (**Figure 6F**). Totally, the inhibition of the MAPK signaling pathway suppressed collagen-mediated cancer progression by regulating both tumor cells and stromal cells.

### Inhibition of the MAPK signaling pathway suppressed collagen-mediated cancer progression *in vivo*

**Figure 7:**
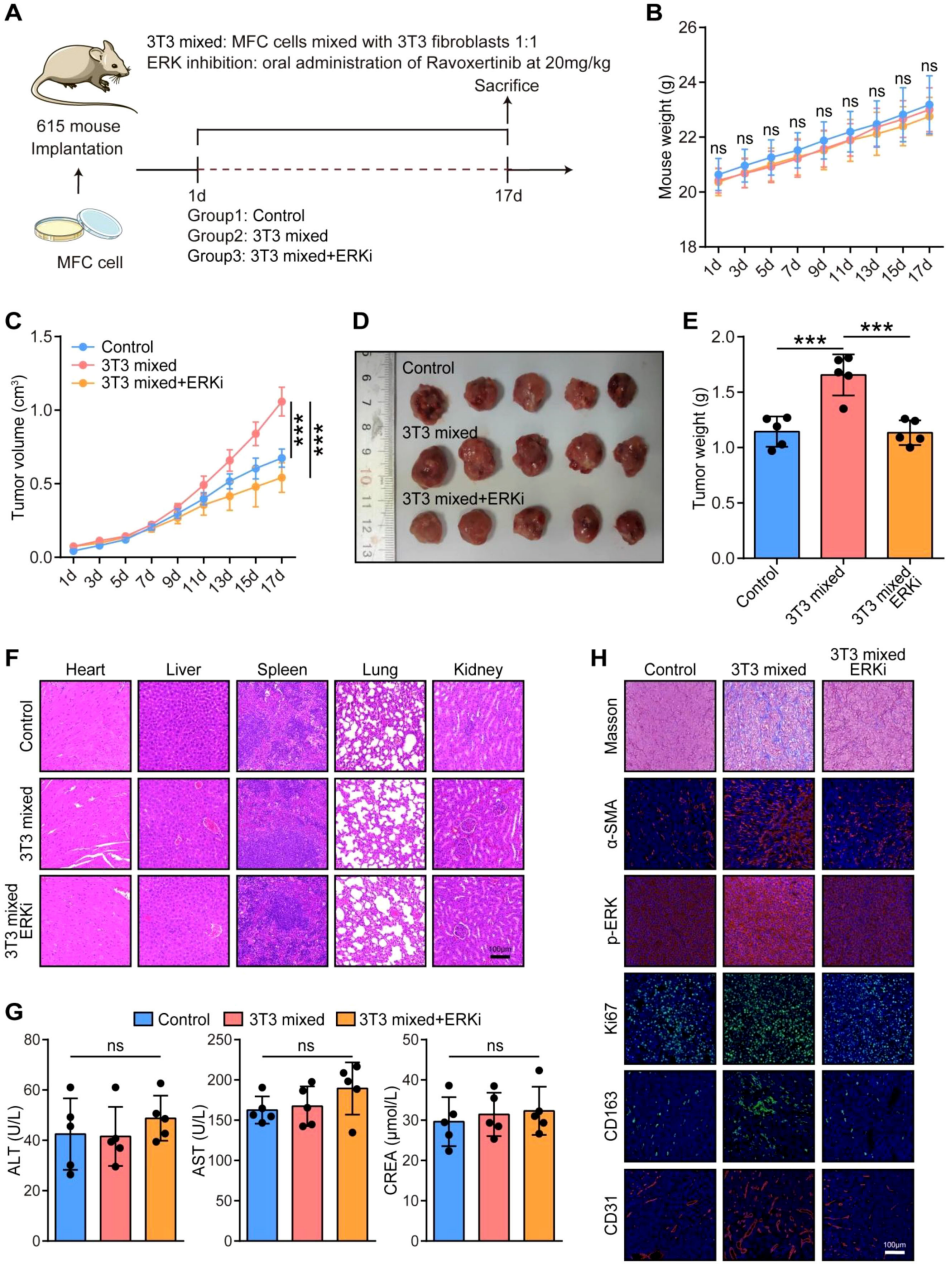
*In vivo* analysis of MAPK signaling pathway blockade in cancer progression. **(A)** Schematic of the establishment of 615 mice bearing MFC cells. **(B)** Effects of Ravoxertinib on 615 mouse weight. Significance determined using ANOVA with Tukey’s multiple comparison test. ns: not significant. n = 5 per group. **(C–E)** Effects of Ravoxertinib on tumor volume and weight in 615 mice bearing MFC cells. Significance determined using ANOVA with Tukey’s multiple comparison test. ***P < 0.001. n = 5 per group. **(F)** Representative images showing structure of heart, liver, spleen, lung, and kidney from mice in different groups. Total original magnification, 200×. Bar = 100 µm. **(G)** Effects of Ravoxertinib on liver and kidney functions in 615 mouse. Significance determined using ANOVA with Tukey’s multiple comparison test. ns: not significant. n = 5 per group. **(H)** Representative images showing the levels of collagen, α-SMA, p-ERK, Ki67, CD163, and CD31 in tumor tissues from mice in different groups. Total original magnification, 200×. Bar = 100 µm.

To further assess the effects of inhibition of the MAPK signaling pathway on cancer progression, we designed an *in vivo* assay. We mixed mouse 3T3 fibroblasts and MFC gastric cancer cells for subcutaneous injection to simulate more collagen deposition in the TME, and Ravoxertinib was injected intraperitoneally to block the MAPK signaling pathway (**Figure 7A**). As the results shown, mixed with fibroblasts and Ravoxertinib treatment did not change mouse weight (**Figure 7B**). However, mixed with fibroblasts significantly accelerated tumor growth, both tumor volume and tumor weight were enhanced remarkably in mice, but Ravoxertinib inhibited tumor progression (**Figures 7C–E**). Furthermore, histological staining of major organs (heart, liver, spleen, lung, and kidney) combined with liver and renal function analyses demonstrated that Ravoxertinib therapy was well-tolerated (**Figures 7F, G**). Histological analysis was performed to check the levels of collagen, α-SMA, p-ERK, Ki67, CD163, and CD31. The results showed that mixed with fibroblasts significantly enhanced collagen deposition and the expression of α-SMA, p-ERK, Ki67, CD163, and CD31 in tumor tissues, but Ravoxertinib therapy reversed these elevations (**Figure 7H**). In summary, these findings suggested that inhibition of the MAPK signaling pathway suppressed collagen-mediated cancer progression *in vivo*.

## Discussion

In the current research, we systematically characterized the clinical and molecular implications of intratumoral collagen deposition in solid cancers, with a focus on gastric cancer. Our findings revealed that elevated collagen levels are significantly associated with poor prognosis. Through comprehensive spatial and molecular analyses, we identified a strong positive correlation between collagen deposition and the abundance of vascular endothelial cells and M2-polarized macrophages within the TME. Furthermore, pathway enrichment analysis demonstrated that collagen-mediated activation of the MAPK signaling pathway plays a pivotal role in promoting tumor cell aggressiveness, angiogenesis, and M2 macrophage polarization. These results collectively highlight the multifaceted role of collagen in shaping the TME and driving tumor progression.

The TME constitutes a complex ecosystem that encompasses a diverse array of stromal and immune cells, extracellular matrix (ECM) components, and signaling molecules (36–38). Among these, collagen, as a major ECM component, has been increasingly recognized for its role in modulating cellular behaviors and signaling pathways (39, 40). Our study adds to this growing body of evidence by uncovering that collagen deposition is closely linked to the recruitment and functional polarization of vascular endothelial cells and M2 macrophages. Specifically, the positive correlation between collagen and M2 macrophages suggests that collagen may facilitate the establishment of an immuno-suppressive TME, which is known to promote tumor immune evasion and progression (41, 42). Similarly, the association with vascular endothelial cells underscores the role of collagen in supporting angiogenesis, a critical process for tumor growth and metastasis (35, 43).

One of the most intriguing results of our study is the identification of the MAPK signaling pathway as a key downstream effector of collagen-mediated TME remodeling. The MAPK signaling pathway is well-known for its involvement in cell proliferation, survival, and differentiation (44, 45). Our data suggest that collagen deposition activates MAPK signaling in tumor cells, endothelial cells, and macrophages, thereby driving multiple pro-tumorigenic processes. For instance, MAPK activation in tumor cells may enhance their invasive potential (46), while in endothelial cells, it may promote angiogenesis (47, 48). In macrophages, MAPK signaling has been implicated in M2 polarization (49), which is consistent with our observation of increased M2 macrophage abundance in high-collagen tumors. Notably, analysis of gastric cancer scRNA−seq data revealed that tumor cells, macrophages, and endothelial cells with high MAPK activity frequently co-upregulated several integrin subunits, specifically ITGB1, ITGB5, and ITGAV (**Supplementary Figures 8A–D**). These subunits form part of known collagen-binding integrins (e.g., α1β1, α2β1, αvβ5) and have been previously linked to MAPK pathway activation (50–52). For instance, a study by Wu et al. showed that COL4A2 promotes glioblastoma vascularization by activating MAPK-ERK signaling through ITGA1/ITGB1 receptors on tumor-associated endothelial cells (52). Based on these observations, we propose ITGB1, ITGB5, and ITGAV as candidate receptors that may mediate collagen−induced MAPK signaling in the gastric cancer TME, positioning them as important targets for future functional validation. These findings provide a mechanistic link between collagen and the functional reprogramming of TME components.

The strong association between collagen deposition and poor clinical outcomes highlights its potential as a prognostic indicator in gastric cancer. Our results suggest that patients with high intratumoral collagen levels may benefit from more aggressive therapeutic strategies. Additionally, the identification of the MAPK signaling pathway as a central mediator of collagen’s effects opens new avenues for targeted therapy. For example, MAPK inhibitors, either alone or in combination with existing therapies (53, 54), could be explored to disrupt collagen-mediated TME remodeling and improve patient outcomes. Furthermore, targeting collagen itself or its downstream effectors may represent a novel therapeutic strategy to counteract tumor progression (34, 55).

Despite the insights our study provides into collagen’s role in gastric cancer, several limitations must be noted. First, although the *in vivo* experiment demonstrated that the MAPK inhibitor Ravoxertinib effectively counteracted the tumor-promoting effect of fibroblast co-injection, the experimental design lacked a control group receiving Ravoxertinib monotherapy. Consequently, the present data cannot conclusively rule out a concomitant general growth-inhibitory effect of Ravoxertinib on MFC cells themselves, which warrants further investigation to delineate the precise mechanism of action. Secondly, the generalizability of our findings to other solid cancers remains to be explored. In addition, our co-transplantation model could not isolate the specific role of collagen from other factors secreted by CAFs. However, supporting *in vitro* evidence confirms that purified collagen alone is sufficient to drive tumor−promoting phenotypes via the MAPK signaling pathway. Moreover, the collagen receptors were various in different cell types, how these collagen receptors linked to the MAPK signaling pathway was perplexing. Future studies should explore whether similar collagen-driven mechanisms exist in other cancers, and whether targeting these pathways holds broader therapeutic relevance.

## Conclusion

In conclusion, our study elucidates the clinical and molecular significance of intratumoral collagen deposition in gastric cancer and other solid cancers. We demonstrate that collagen accumulation is associated with an immunosuppressive and pro-angiogenic TME, driven in part by MAPK signaling pathway activation. These findings not only enhance our understanding of the complex interplay between ECM components and TME dynamics but also provide a rationale for developing novel therapeutic strategies targeting collagen and its downstream signaling pathways. Future research should focus on translating these insights into clinical applications to improve outcomes for patients with gastric and other solid cancers.

## Data Availability

The original contributions presented in the study are included in the article/**Supplementary Material**. Further inquiries can be directed to the corresponding authors.

## References

[1] Maher M Castilho M Yue Z Glattauer V Hughes TC Ramshaw JAM . Shaping collagen for engineering hard tissues: Towards a printomics approach. Acta Biomater. (2021) 131:41–61. doi: 10.1016/j.actbio.2021.06.035, PMID: 34192571

[2] Wang H . The potential of collagen treatment for comorbid diseases. Polym (Basel). (2023) 15. doi: 10.3390/polym15193999, PMID: 37836047 PMC10574914

[3] Zhang KW Liu SY Jia Y Zou ML Teng YY Chen ZH . Insight into the role of DPP-4 in fibrotic wound healing. BioMed Pharmacother. (2022) 151:113143. doi: 10.1016/j.biopha.2022.113143, PMID: 35643071

[4] Wang Y Jiao L Qiang C Chen C Shen Z Ding F . The role of matrix metalloproteinase 9 in fibrosis diseases and its molecular mechanisms. BioMed Pharmacother. (2024) 171:116116. doi: 10.1016/j.biopha.2023.116116, PMID: 38181715

[5] Wang K Ning S Zhang S Jiang M Huang Y Pei H . Extracellular matrix stiffness regulates colorectal cancer progression via HSF4. J Exp Clin Cancer Res. (2025) 44:30. doi: 10.1186/s13046-025-03297-8, PMID: 39881364 PMC11780783

[6] Yang Y Sun H Yu H Wang L Gao C Mei H . Tumor-associated-fibrosis and active collagen-CD44 axis characterize a poor-prognosis subtype of gastric cancer and contribute to tumor immunosuppression. J Transl Med. (2025) 23:123. doi: 10.1186/s12967-025-06070-9, PMID: 39871345 PMC11773867

[7] Peng DH Rodriguez BL Diao L Chen L Wang J Byers LA . Collagen promotes anti-PD-1/PD-L1 resistance in cancer through LAIR1-dependent CD8(+) T cell exhaustion. Nat Commun. (2020) 11:4520. doi: 10.1038/s41467-020-18298-8, PMID: 32908154 PMC7481212

[8] Zhang H Lin H Wang J Wu D Wang Q Mei J . Tumor-stroma contributes to immunotherapeutic resistance in non-small cell lung cancer via SEMA3C-mediated immunosuppressive tumor microenvironment. Transl Oncol. (2026) 65:102679. doi: 10.1016/j.tranon.2026.102679, PMID: 41558146 PMC12857187

[9] Wu G Pan B Shi H Yi Y Zheng X Ma H . Neutrophils’ dual role in cancer: from tumor progression to immunotherapeutic potential. Int Immunopharmacol. (2024) 140:112788. doi: 10.1016/j.intimp.2024.112788, PMID: 39083923

[10] Mei J Cai Y Xu R Li Q Chu J Luo Z . Conserved immuno-collagenic subtypes predict response to immune checkpoint blockade. Cancer Commun (Lond). (2024) 44:554–75. doi: 10.1002/cac2.12538, PMID: 38507505 PMC11110954

[11] Hartmann N Giese NA Giese T Poschke I Offringa R Werner J . Prevailing role of contact guidance in intrastromal T-cell trapping in human pancreatic cancer. Clin Cancer Res. (2014) 20:3422–33. doi: 10.1158/1078-0432.CCR-13-2972, PMID: 24763614

[12] Vijver SV Singh A Mommers-Elshof E Meeldijk J Copeland R Boon L . Collagen fragments produced in cancer mediate T cell suppression through leukocyte-associated immunoglobulin-like receptor 1. Front Immunol. (2021) 12:733561. doi: 10.3389/fimmu.2021.733561, PMID: 34691040 PMC8529287

[13] O’Connor RS Hao X Shen K Bashour K Akimova T Hancock WW . Substrate rigidity regulates human T cell activation and proliferation. J Immunol. (2012) 189:1330–9. doi: 10.4049/jimmunol.1102757, PMID: 22732590 PMC3401283

[14] Liu Y Yao X Zhao Y Fang D Shi L Yang L . Mechanotransduction in response to ECM stiffening impairs cGAS immune signaling in tumor cells. Cell Rep. (2023) 42:113213. doi: 10.1016/j.celrep.2023.113213, PMID: 37804510

[15] Pusztai L Yau C Wolf DM Han HS Du L Wallace AM . Durvalumab with olaparib and paclitaxel for high-risk HER2-negative stage II/III breast cancer: Results from the adaptively randomized I-SPY2 trial. Cancer Cell. (2021) 39:989–98.e5. doi: 10.1016/j.ccell.2021.05.009, PMID: 34143979 PMC11064785

[16] Wolf DM Yau C Wulfkuhle J Brown-Swigart L Gallagher RI Lee PRE . Redefining breast cancer subtypes to guide treatment prioritization and maximize response: Predictive biomarkers across 10 cancer therapies. Cancer Cell. (2022) 40:609–23.e6. doi: 10.1016/j.ccell.2022.05.005, PMID: 35623341 PMC9426306

[17] Kim ST Cristescu R Bass AJ Kim KM Odegaard JI Kim K . Comprehensive molecular characterization of clinical responses to PD-1 inhibition in metastatic gastric cancer. Nat Med. (2018) 24:1449–58. doi: 10.1038/s41591-018-0101-z, PMID: 30013197

[18] Jung H Kim HS Kim JY Sun JM Ahn JS Ahn MJ . DNA methylation loss promotes immune evasion of tumours with high mutation and copy number load. Nat Commun. (2019) 10:4278. doi: 10.1038/s41467-019-12159-9, PMID: 31537801 PMC6753140

[19] Hugo W Zaretsky JM Sun L Song C Moreno BH Hu-Lieskovan S . Genomic and transcriptomic features of response to anti-PD-1 therapy in metastatic melanoma. Cell. (2016) 165:35–44. doi: 10.1016/j.cell.2016.02.065, PMID: 26997480 PMC4808437

[20] Rose TL Weir WH Mayhew GM Shibata Y Eulitt P Uronis JM . Fibroblast growth factor receptor 3 alterations and response to immune checkpoint inhibition in metastatic urothelial cancer: a real world experience. Br J Cancer. (2021) 125:1251–60. doi: 10.1038/s41416-021-01488-6, PMID: 34294892 PMC8548561

[21] Blenman KRM Marczyk M Karn T Qing T Li X Gunasekharan V . Predictive markers of response to neoadjuvant durvalumab with nab-paclitaxel and dose-dense doxorubicin/cyclophosphamide in basal-like triple-negative breast cancer. Clin Cancer Res. (2022) 28:2587–97. doi: 10.1158/1078-0432.CCR-21-3215, PMID: 35377948 PMC9464605

[22] Ritchie ME Phipson B Wu D Hu Y Law CW Shi W . limma powers differential expression analyses for RNA-sequencing and microarray studies. Nucleic Acids Res. (2015) 43:e47. doi: 10.1093/nar/gkv007, PMID: 25605792 PMC4402510

[23] Cai Y Ji W Sun C Xu R Chen X Deng Y . Interferon-induced transmembrane protein 3 shapes an inflamed tumor microenvironment and identifies immuno-hot tumors. Front Immunol. (2021) 12:704965. doi: 10.3389/fimmu.2021.704965, PMID: 34456915 PMC8385493

[24] Mei J Fu Z Cai Y Song C Zhou J Zhu Y . SECTM1 is upregulated in immuno-hot tumors and predicts immunotherapeutic efficacy in multiple cancers. iScience. (2023) 26:106027. doi: 10.1016/j.isci.2023.106027, PMID: 36818292 PMC9932126

[25] Mei J Cai Y Wang H Xu R Zhou J Lu J . Formin protein DIAPH1 positively regulates PD-L1 expression and predicts the therapeutic response to anti-PD-1/PD-L1 immunotherapy. Clin Immunol. (2023) 246:109204. doi: 10.1016/j.clim.2022.109204, PMID: 36503156

[26] Cai Y Cheng Y Wang Z Li L Qian Z Xia W . A novel metabolic subtype with S100A7 high expression represents poor prognosis and immuno-suppressive tumor microenvironment in bladder cancer. BMC Cancer. (2023) 23:725. doi: 10.1186/s12885-023-11182-w, PMID: 37543645 PMC10403905

[27] Kumar V Ramnarayanan K Sundar R Padmanabhan N Srivastava S Koiwa M . Single-cell atlas of lineage states, tumor microenvironment, and subtype-specific expression programs in gastric cancer. Cancer Discov. (2022) 12:670–91. doi: 10.1158/2159-8290.CD-21-0683, PMID: 34642171 PMC9394383

[28] Korsunsky I Millard N Fan J Slowikowski K Zhang F Wei K . Fast, sensitive and accurate integration of single-cell data with Harmony. Nat Methods. (2019) 16:1289–96. doi: 10.1038/s41592-019-0619-0, PMID: 31740819 PMC6884693

[29] Tsuyuzaki K Sato H Sato K Nikaido I . Benchmarking principal component analysis for large-scale single-cell RNA-sequencing. Genome Biol. (2020) 21:9. doi: 10.1186/s13059-019-1900-3, PMID: 31955711 PMC6970290

[30] Shekhar K Lapan SW Whitney IE Tran NM Macosko EZ Kowalczyk M . Comprehensive classification of retinal bipolar neurons by single-cell transcriptomics. Cell. (2016) 166:1308–23.e30. doi: 10.1016/j.cell.2016.07.054, PMID: 27565351 PMC5003425

[31] Kotliarov Y Sparks R Martins AJ Mule MP Lu Y Goswami M . Broad immune activation underlies shared set point signatures for vaccine responsiveness in healthy individuals and disease activity in patients with lupus. Nat Med. (2020) 26:618–29. doi: 10.1038/s41591-020-0769-8, PMID: 32094927 PMC8392163

[32] Valastyan S Weinberg RA . Tumor metastasis: molecular insights and evolving paradigms. Cell. (2011) 147:275–92. doi: 10.1016/j.cell.2011.09.024, PMID: 22000009 PMC3261217

[33] Mei J Jiang G Chen Y Xu Y Wan Y Chen R . HLA class II molecule HLA-DRA identifies immuno-hot tumors and predicts the therapeutic response to anti-PD-1 immunotherapy in NSCLC. BMC Cancer. (2022) 22:738. doi: 10.1186/s12885-022-09840-6, PMID: 35794593 PMC9258174

[34] Mei J Chu J Yang K Luo Z Yang J Xu J . Angiotensin receptor blocker attacks armored and cold tumors and boosts immune checkpoint blockade. J Immunother Cancer. (2024) 12. doi: 10.1136/jitc-2024-009327, PMID: 39244215 PMC11418576

[35] Mei J Yang K Zhang X Luo Z Tian M Fan H . Intratumoral collagen deposition supports angiogenesis suggesting anti-angiogenic therapy in armored and cold tumors. Adv Sci (Weinh). (2025) 12:e2409147. doi: 10.1002/advs.202409147, PMID: 39823457 PMC11904994

[36] Rodriguez-Bejarano OH Parra-Lopez C Patarroyo MA . A review concerning the breast cancer-related tumour microenvironment. Crit Rev Oncol Hematol. (2024) 199:104389. doi: 10.1016/j.critrevonc.2024.104389, PMID: 38734280

[37] Zhang H Cao X Gui R Li Y Zhao X Mei J . Mesenchymal Stem/Stromal cells in solid tumor Microenvironment: Orchestrating NK cell remodeling and therapeutic insights. Int Immunopharmacol. (2024) 142:113181. doi: 10.1016/j.intimp.2024.113181, PMID: 39305890

[38] Chen L Lin A Tang B Li K Qian Y Liu X . The future of cancer therapy: nanomaterials and tumor microenvironment. iMetaMed. (2025) 1. doi: 10.1002/imm3.70007

[39] Lo Buglio G Lo Cicero A Campora S Ghersi G . The multifaced role of collagen in cancer development and progression. Int J Mol Sci. (2024) 25. doi: 10.3390/ijms252413523, PMID: 39769286 PMC11678882

[40] Zhang X Zhang X Huang W Ge X . The role of heat shock proteins in the regulation of fibrotic diseases. BioMed Pharmacother. (2021) 135:111067. doi: 10.1016/j.biopha.2020.111067, PMID: 33383375

[41] Pan B Shen S Zhao J Zhang Z Ye D Zhang X . LAIR1 promotes hepatocellular carcinoma cell metastasis and induces M2-macrophage infiltration through activating AKT-IKKbeta-p65 axis. Mol Carcinog. (2024) 63:1827–41. doi: 10.1002/mc.23776, PMID: 39016636

[42] Bachy S Wu Z Gamradt P Thierry K Milani P Chlasta J . betaig-h3-structured collagen alters macrophage phenotype and function in pancreatic cancer. iScience. (2022) 25:103758. doi: 10.1016/j.isci.2022.103758, PMID: 35146384 PMC8816720

[43] Dartora VFC Carney R Wang A Qiu P Panitch A . Extracellular matrix ligands modulate the endothelial progenitor cell secretome for enhanced angiogenesis. Acta Biomater. (2025) 195:240–55. doi: 10.1016/j.actbio.2025.02.028, PMID: 39954753 PMC12590308

[44] Garcia-Hernandez L Garcia-Ortega MB Ruiz-Alcala G Carrillo E Marchal JA Garcia MA . The p38 MAPK components and modulators as biomarkers and molecular targets in cancer. Int J Mol Sci. (2021) 23. doi: 10.3390/ijms23010370, PMID: 35008796 PMC8745478

[45] Xia M Zhang Y Wu H Zhang Q Liu Q Li G . Forsythoside B attenuates neuro-inflammation and neuronal apoptosis by inhibition of NF-kappaB and p38-MAPK signaling pathways through activating Nrf2 post spinal cord injury. Int Immunopharmacol. (2022) 111:109120. doi: 10.1016/j.intimp.2022.109120, PMID: 35944463

[46] Sarg NH Zaher DM Abu Jayab NN Mostafa SH Ismail HH Omar HA . The interplay of p38 MAPK signaling and mitochondrial metabolism, a dynamic target in cancer and pathological contexts. Biochem Pharmacol. (2024) 225:116307. doi: 10.1016/j.bcp.2024.116307, PMID: 38797269

[47] Li S Mai H Zhu Y Li G Sun J Li G . MicroRNA-4500 inhibits migration, invasion, and angiogenesis of breast cancer cells via RRM2-dependent MAPK signaling pathway. Mol Ther Nucleic Acids. (2020) 21:278–89. doi: 10.1016/j.omtn.2020.04.018, PMID: 32615527 PMC7330432

[48] Blucher RO Lim RS Ritchie ME Western PS . VEGF-dependent testicular vascularisation involves MEK1/2 signalling and the essential angiogenesis factors, SOX7 and SOX17. BMC Biol. (2024) 22:222. doi: 10.1186/s12915-024-02003-y, PMID: 39354506 PMC11445939

[49] Dong S Li X Chen Z Shi H Wang Z Zhou W . MMP28 recruits M2-type tumor-associated macrophages through MAPK/JNK signaling pathway-dependent cytokine secretion to promote the Malignant progression of pancreatic cancer. J Exp Clin Cancer Res. (2025) 44:60. doi: 10.1186/s13046-025-03321-x, PMID: 39972459 PMC11837641

[50] Gu L Jin X Liang H Yang C Zhang Y . Upregulation of CSNK1A1 induced by ITGB5 confers to hepatocellular carcinoma resistance to sorafenib *in vivo* by disrupting the EPS15/EGFR complex. Pharmacol Res. (2023) 192:106789. doi: 10.1016/j.phrs.2023.106789, PMID: 37149115

[51] Huang M Wang Y Wang Z Qin Q Zhang H Liu S . miR-134-5p inhibits osteoclastogenesis through a novel miR-134-5p/Itgb1/MAPK pathway. J Biol Chem. (2022) 298:102116. doi: 10.1016/j.jbc.2022.102116, PMID: 35691339 PMC9257423

[52] Lee DKC Chen K Loke R Li X Liubart D Saffi GT . ITGAV and SMAD4 influence the progression and clinical outcome of pancreatic ductal adenocarcinoma. Mol Oncol. (2025) 19:3342–59. doi: 10.1002/1878-0261.70080, PMID: 40739706 PMC12591305

[53] Rozeman EA Versluis JM Sikorska K Hoefsmit EP Dimitriadis P Rao D . IMPemBra: a phase 2 study comparing pembrolizumab with intermittent/short-term dual MAPK pathway inhibition plus pembrolizumab in patients with melanoma harboring the BRAFV600 mutation. J Immunother Cancer. (2023) 11. doi: 10.1136/jitc-2023-006821, PMID: 37479483 PMC10364170

[54] Ribas A Algazi A Ascierto PA Butler MO Chandra S Gordon M . PD-L1 blockade in combination with inhibition of MAPK oncogenic signaling in patients with advanced melanoma. Nat Commun. (2020) 11:6262. doi: 10.1038/s41467-020-19810-w, PMID: 33288749 PMC7721806

[55] Narra K Mullins SR Lee HO Strzemkowski-Brun B Magalong K Christiansen VJ . Phase II trial of single agent Val-boroPro (Talabostat) inhibiting Fibroblast Activation Protein in patients with metastatic colorectal cancer. Cancer Biol Ther. (2007) 6:1691–9. doi: 10.4161/cbt.6.11.4874, PMID: 18032930

