# Correction: Collagen-mediated pro-tumorigenic MAPK activation drives stromal-immune reprogramming in solid cancers

**DOI:** 10.3389/fimmu.2026.1821819

**Published:** 2026-03-09

**Authors:** Junli Ding, Hongxin Lin, Hanfang Fan, Liangfeng Xu, Haihua Zhou, Huning Jiang, Zeyu Wang, Tiansong Xia, Yun Cai, Yichao Zhu, Junying Xu

**Affiliations:** 1 Departments of Oncology, The Affiliated Wuxi People’s Hospital of Nanjing Medical University, Wuxi, Jiangsu, China; 2 Wuxi Medical Center, Nanjing Medical University, Wuxi, Jiangsu, China; 3 Department of General Surgery, The First Affiliated Hospital with Nanjing Medical University, Nanjing, Jiangsu, China; 4 The First Clinical Medicine College, Nanjing Medical University, Nanjing, Jiangsu, China; 5 Department of Physiology, School of Basic Medical Sciences, Nanjing Medical University, Nanjing, Jiangsu, China; 6 Department of Gastroenterology, Sheyang County People’s Hospital, Yancheng, Jiangsu, China; 7 Department of General Surgery, The Affiliated Taizhou People’s Hospital of Nanjing Medical University, Taizhou, Jiangsu, China; 8 Taizhou School of Clinical Medicine, Nanjing Medical University, Taizhou, Jiangsu, China; 9 Central Laboratory, Jintan Affiliated Hospital of Jiangsu University, Changzhou, Jiangsu, China

**Keywords:** collagen deposition, M2 macrophage, MAPK, microvessel density, pan-cancer

Affiliations “Department of Physiology, School of Basic Medical Sciences, Nanjing Medical University, Nanjing, Jiangsu, China; Department of General Surgery, The Affiliated Taizhou People’s Hospital of Nanjing Medical University, Taizhou, Jiangsu, China” were omitted for author “Yichao Zhu”. These affiliations have now been added for author “Yichao Zhu”.

The previously listed affiliations for author “Yichao Zhu” should be disregarded and replaced with the ones stated above. The affiliation of other authors remains unchanged.

The original version of this article has been updated.

