# Supplementary material for: Collagen-mediated pro-tumorigenic MAPK activation drives stromal-immune reprogramming in solid cancers
Source: Front Immunol. 2026 Feb 24;17:1744126. doi: 10.3389/fimmu.2026.1744126 (PMC13006797; doi:10.3389/fimmu.2026.1744126)
Supplement: Supplementary file 1 [file DataSheet1.docx]

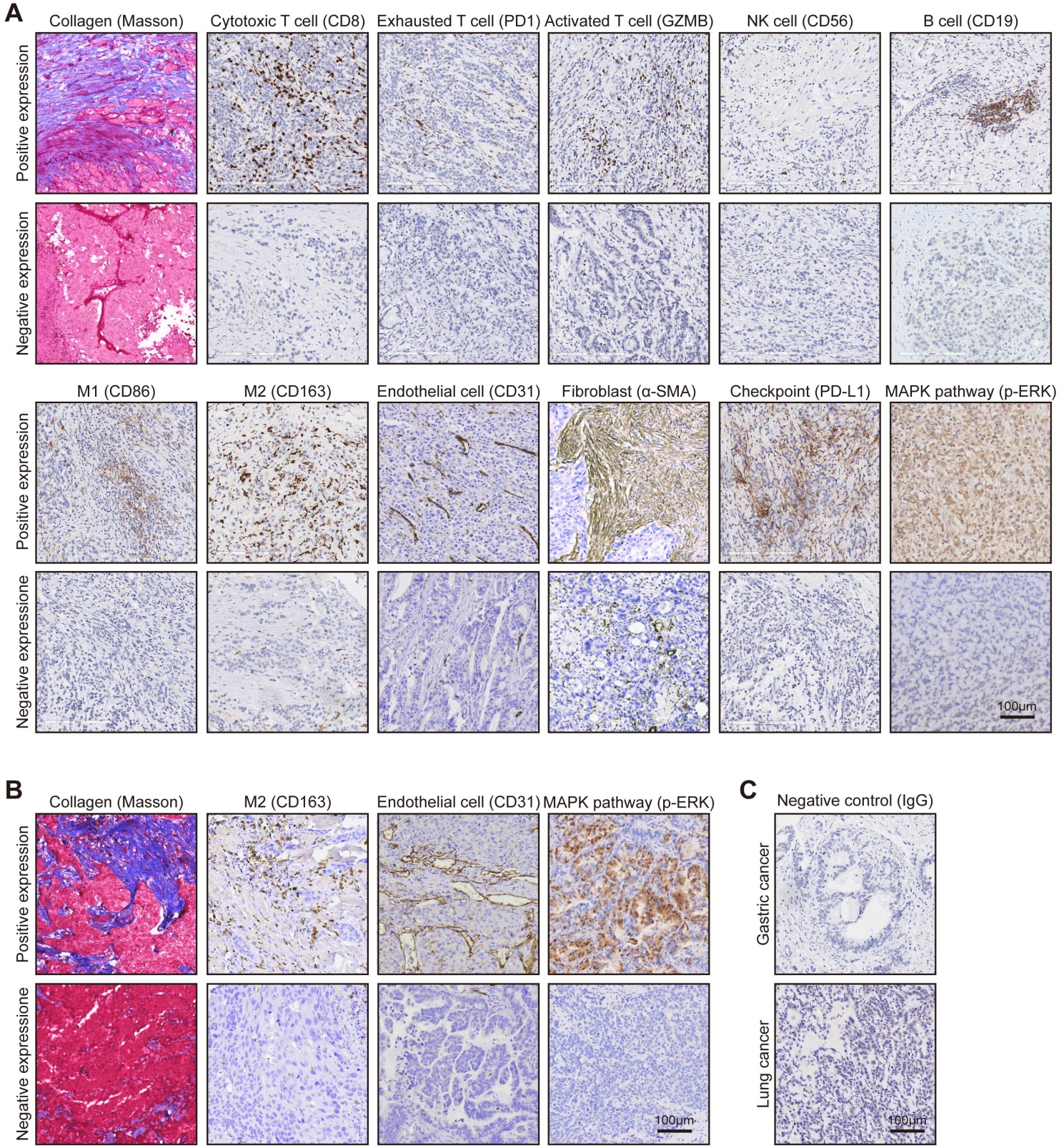


**Supplementary Figure 1. Representative images showing the staining markers.** (A) Masson staining and various IHC markers in gastric cancer. (B) Masson staining and various IHC markers in lung cancer. (C) Negative control in gastric and lung cancer. Bar = 100 µm.


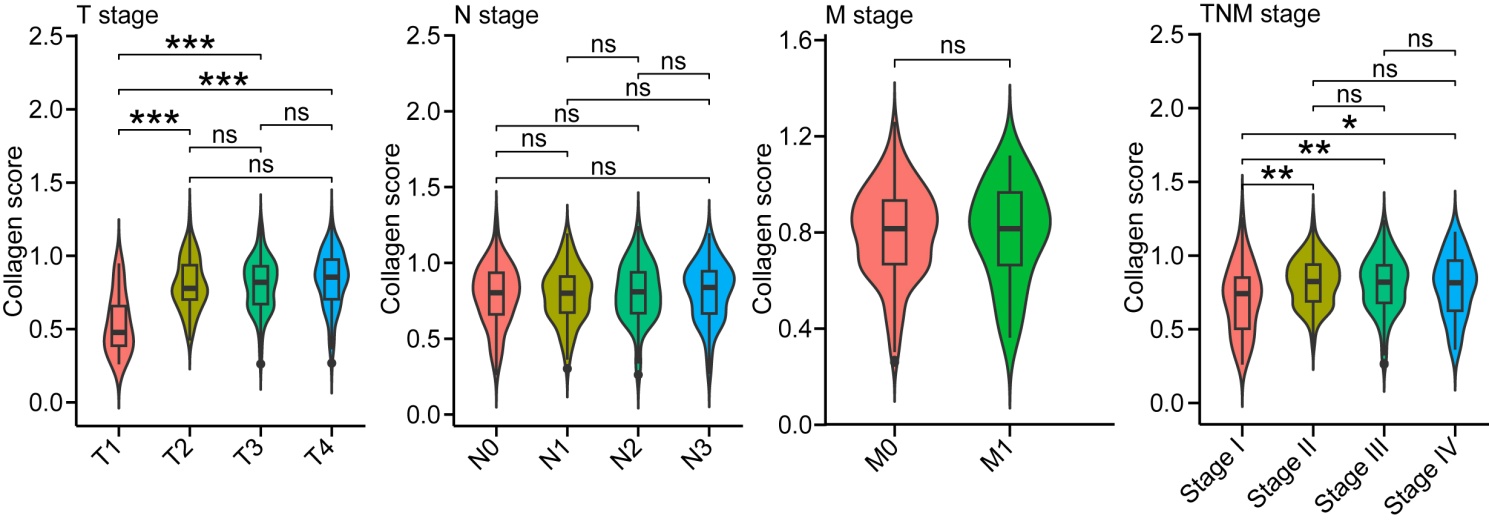


**Supplementary Figure 2. Difference in collagen score in tumor tissues with various clinical stages.** Significance was calculated using the one-way analysis of variance test with multiple comparisons or the student t test. ns: non-significance, *P<0.05, **P<0.01, ***P<0.001.


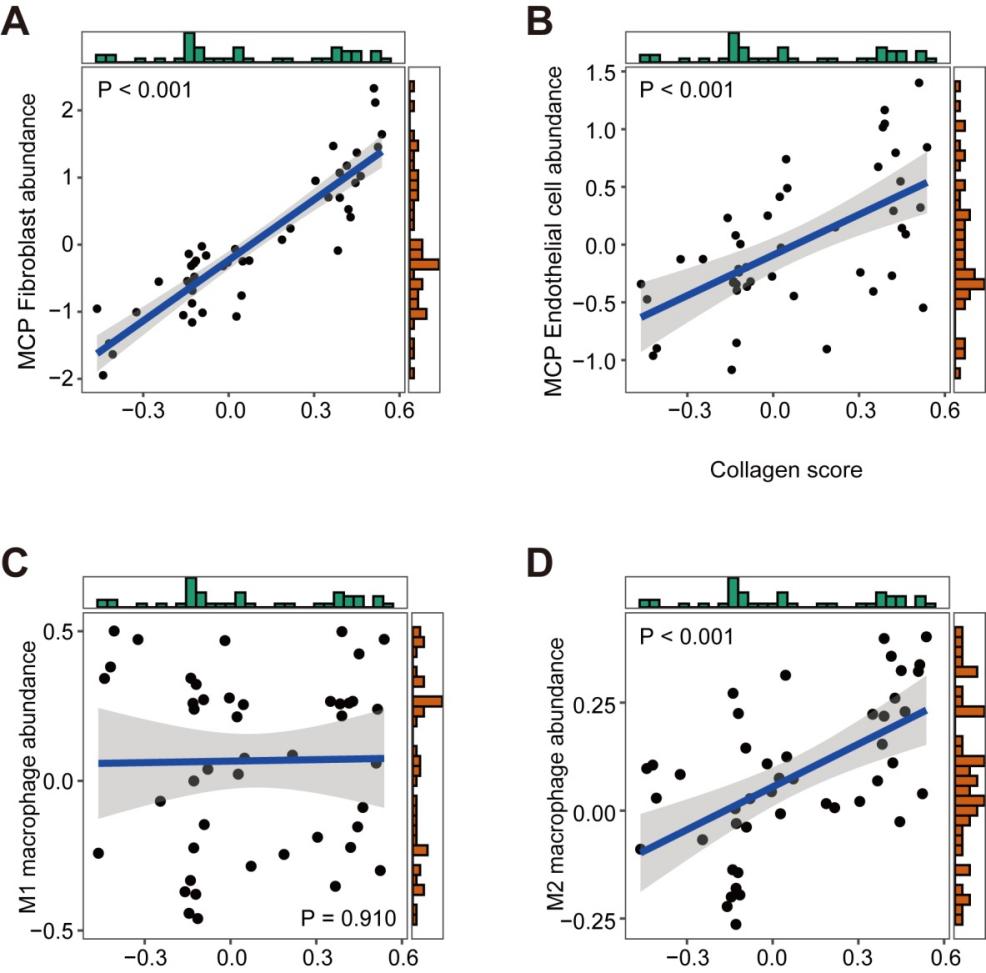


**Supplementary Figure 3.** **Correlations between collagen deposition and cell fractions in the PRJEB25780 dataset.** (A) Fibroblasts estimated by the MCP-counter. (B) Endothelial cells estimated by the MCP-counter. (C) M1 macrophage. (D) M1 macrophage. Significance was calculated using the Pearson test.


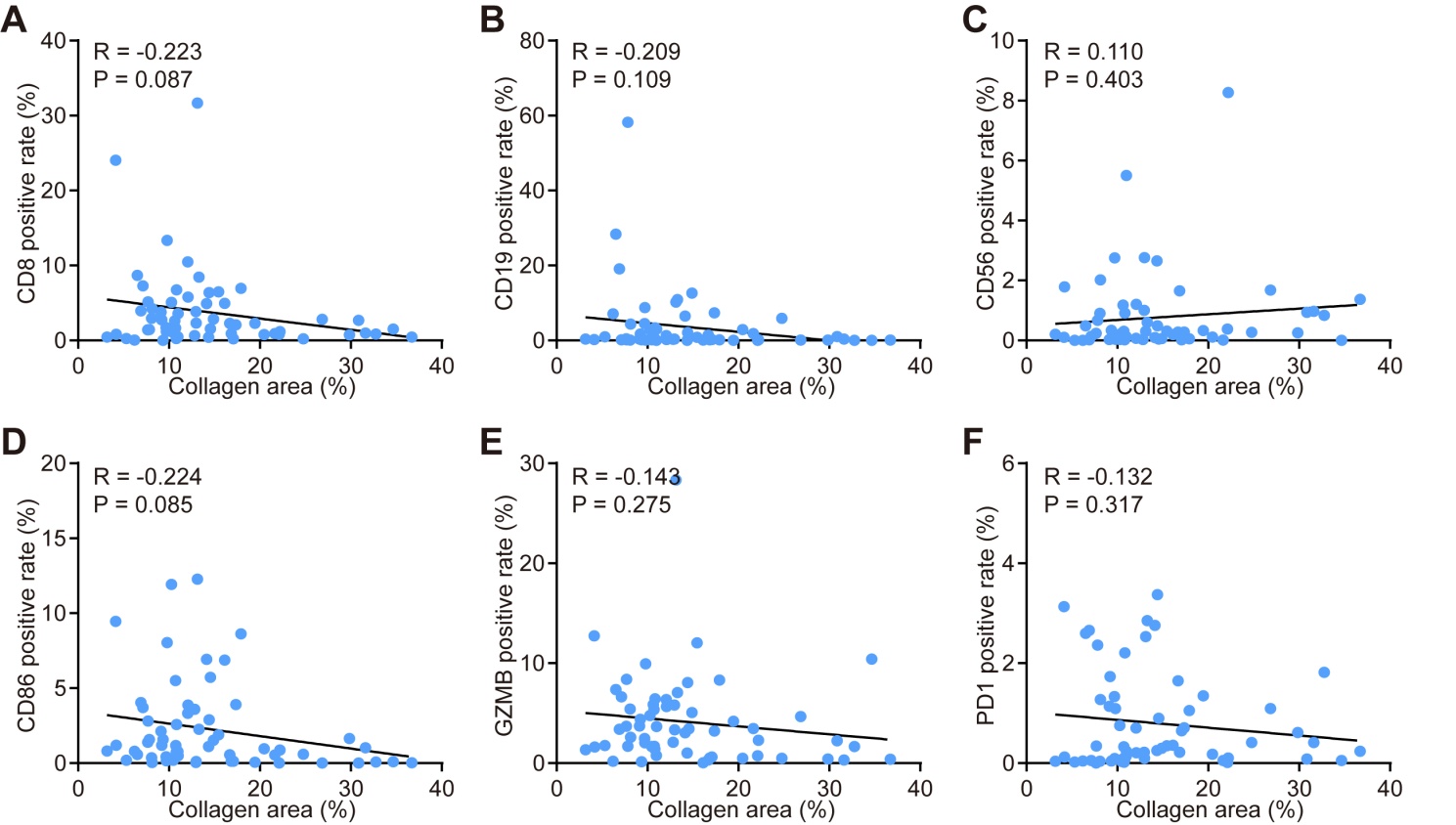


**Supplementary Figure 4. Correlations between collagen deposition and cell fractions in the in-house cohort.** (A) CD8+ T cells (CD8). (B) B cells (CD19). (C) NK cells (CD56). (D) M1 macrophages (CD86). (E) Activated T cells (GZMB). (F) Exhausted T cells (PD1). Significance was calculated using the Pearson test.


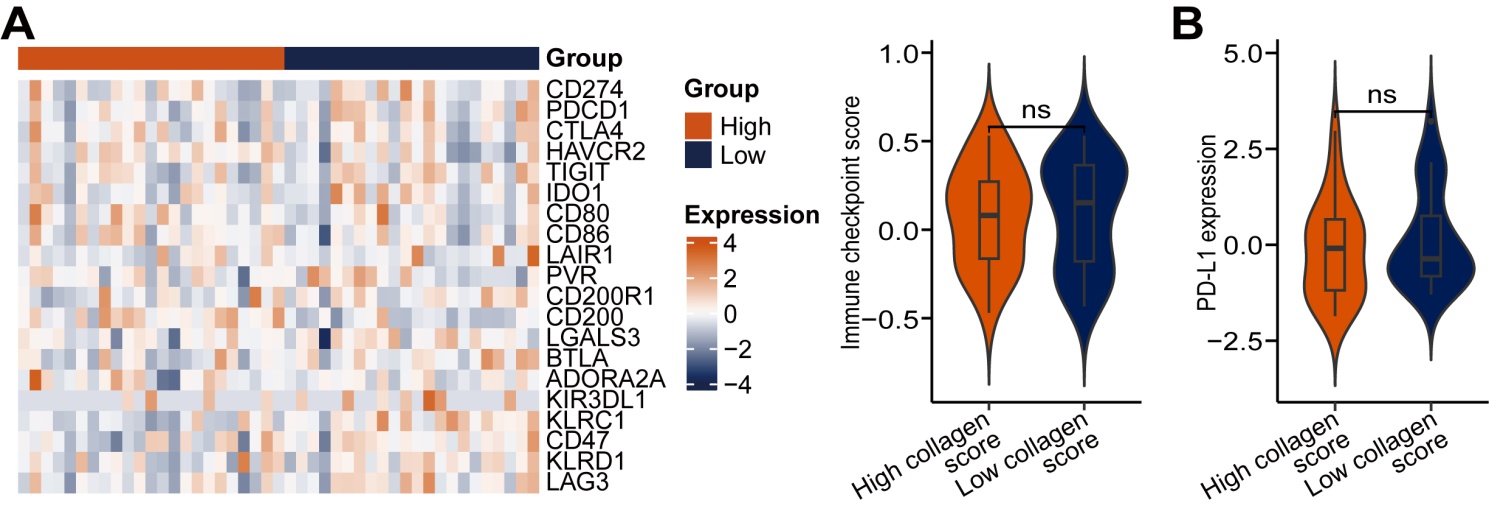


**Supplementary Figure 5.** **Correlations between collagen deposition and immune checkpoints in the PRJEB25780 dataset.** (A) Heatmap showing the expression of immune checkpoints in low and high collagen scores groups and GSVA analysis. (B) Difference in PD-L1 expression in low and high collagen scores groups. Significance was calculated using the student t test. ns: non-significance.


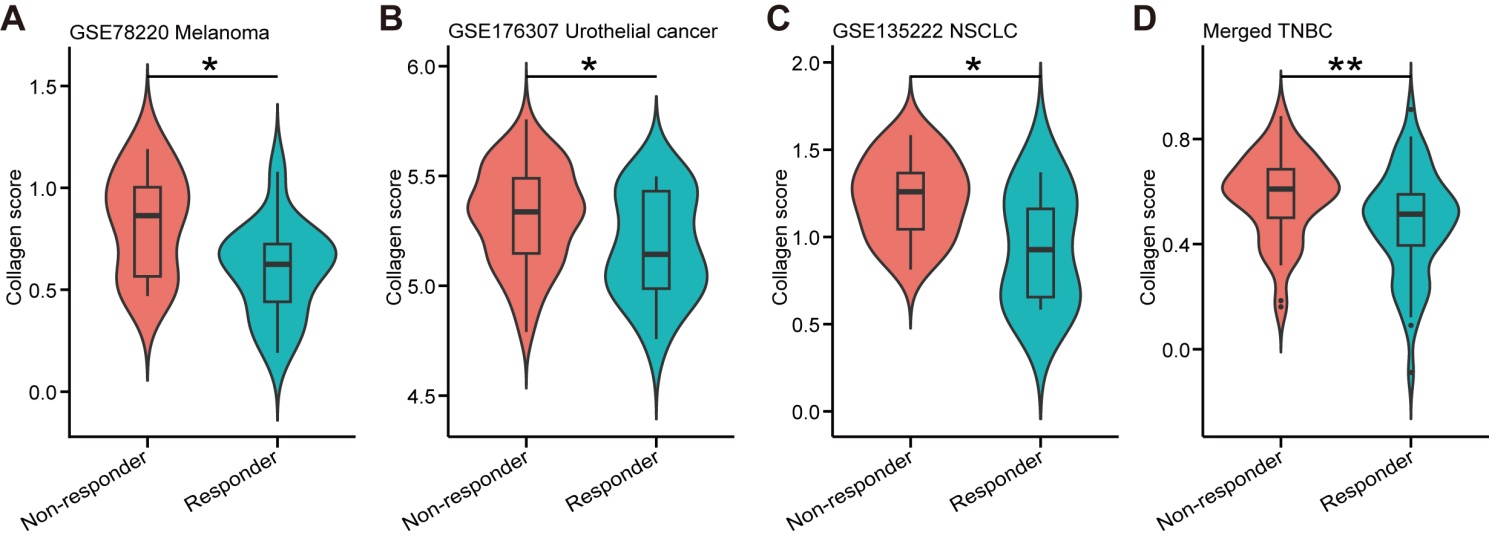


**Supplementary Figure 6. Pan-cancer analysis of the predictive values of collagen score in public immunotherapy datasets.** (A) The GSE78220 dataset. (B) The GSE176307 dataset. (C) The GSE13522 dataset. (D) The merged TNBC dataset. Significance was calculated using the student t test. **P<0.01, ***P<0.001.


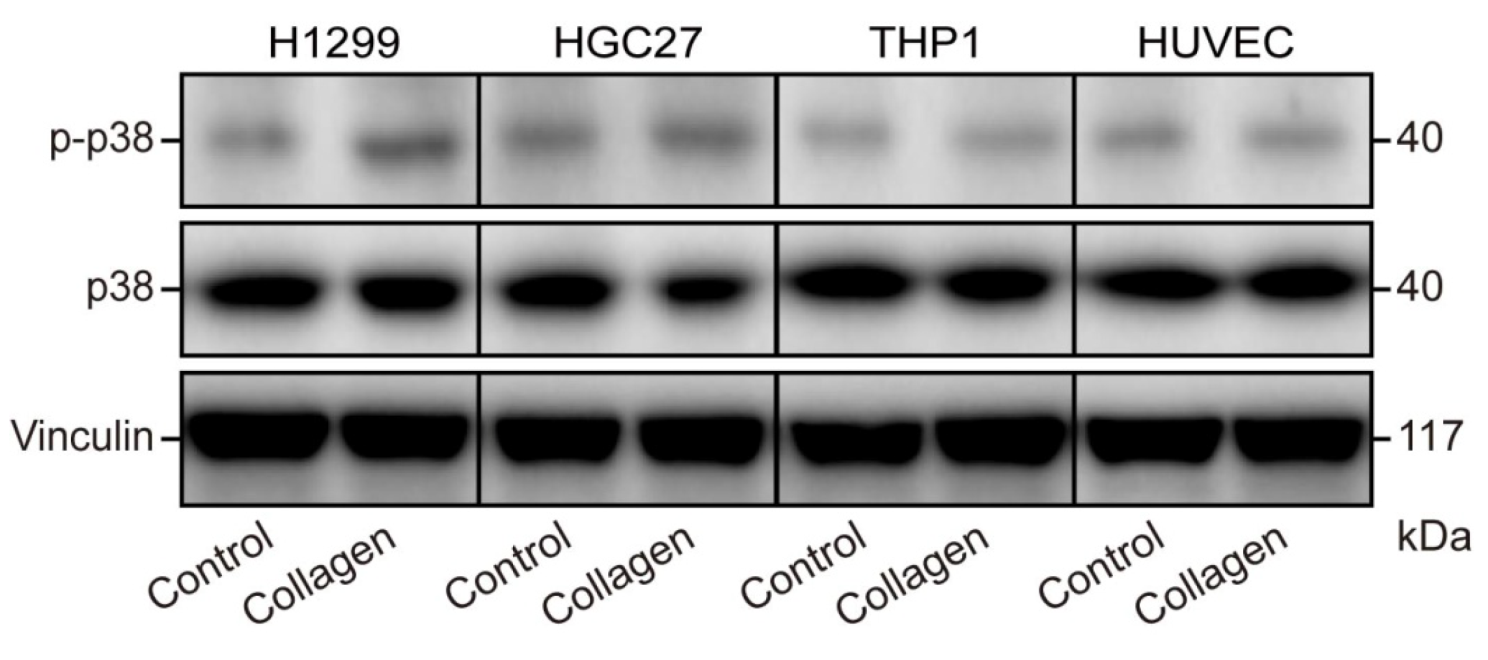


**Supplementary Figure 7. Activated effects of collagen on p38 and p-p38 in H1299, HGC27, THP-1, and HUVEC cells.**


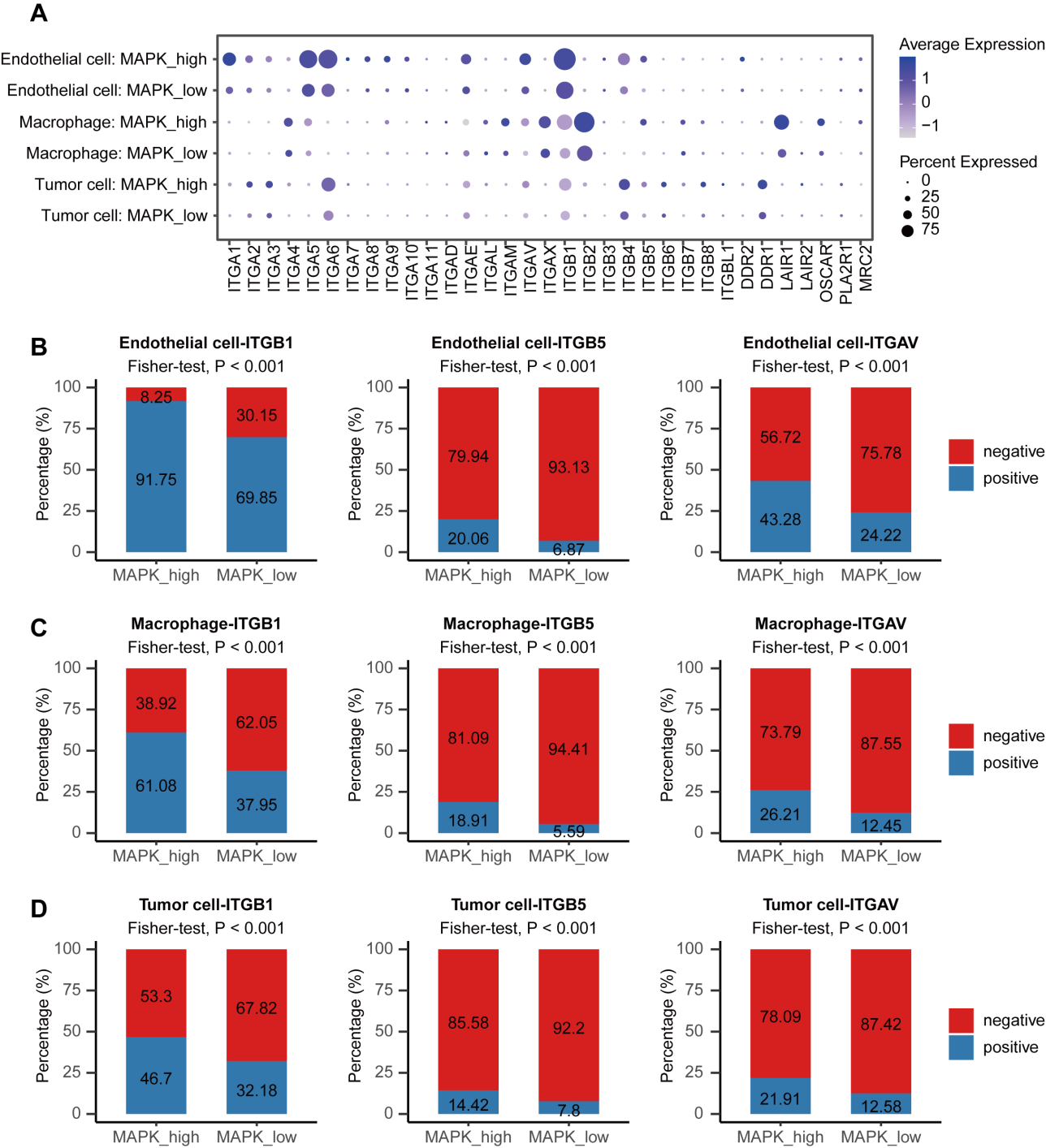


**Supplementary Figure 8 . Expression of collagen receptors in tumor cells, macrophages, and endothelial cells with high or low MAPK pathway activity.** (A) Bubble plot showing the expression levels of collagen receptors across tumor cells, macrophages, and endothelial cells stratified by high vs. low MAPK signaling. (B-D) Proportion of ITGB1-, ITGB5-, and ITGAV- positive cells versus negative cells in endothelial cells (B), macrophages (C), and tumor cells (D) with high or low MAPK signaling. Differences between groups were assessed using Fisher’s exact test.

**Supplementary Table 1. List of collagen‑related genes included in the collagen score.**

| Subject | Genes |
| --- | --- |
| Collagen score | COL1A1, COL1A2, COL2A1, COL3A1, COL4A1, COL4A2, COL4A4, COL4A5, COL4A6, COL5A1, COL5A2, COL5A3, COL6A1, COL6A2, COL6A3, COL6A6, COL7A1, COL8A1, COL8A2, COL9A1, COL9A2, COL9A3, COL10A1, COL11A1, COL11A1, COL11A2, COL12A1, COL12A1, COL13A1, COL14A1, COL15A1, COL16A1, COL17A1, COL18A1, COL19A1, COL20A1, COL21A1, COL22A1, COL23A1, COL24A1, COL25A1, COL27A1, COL28A1, COL29A1 |

**Supplementary Table 2. GSEA analysis of activities of molecular pathways between low and high collagen scores groups in the TCGA dataset.**

| **Description** | **NES** | **P value** |
| --- | --- | --- |
| MAPK signaling pathway | 1.483 | 6.77E-10 |
| Wnt-betacatenin signaling pathway | 1.487 | 2.44E-07 |
| TGFbeta-SMAD signaling pathway | 1.678 | 4.19E-06 |
| HEDGEHOG signaling pathway | 1.459 | 0.000253 |
| Hypoxia | 1.361 | 0.003978 |
| WNT signaling pathway | 1.253 | 0.009341 |
| PI3K-Akt signaling pathway | 1.416 | 0.013156 |
| YAP1 UP | 1.406 | 0.043659 |
| YAP conserved signature | 1.393 | 0.043744 |
| YAP1 DN | 1.406 | 0.050483 |
| HIPPI-YAP signaling pathway | 1.325 | 0.095933 |
| JAK-STAT signaling pathway | 1.159 | 0.217782 |
| NOTCH signaling pathway | 1.058 | 0.310689 |
| NFKB signaling pathway | 1.025 | 0.445554 |
